# Discovery, characterization, and engineering of an advantageous *Streptomyces* host for heterologous expression of natural product biosynthetic gene clusters

**DOI:** 10.1186/s12934-024-02416-y

**Published:** 2024-05-24

**Authors:** Evaldas Klumbys, Wei Xu, Lokanand Koduru, Elena Heng, Yifeng Wei, Fong Tian Wong, Huimin Zhao, Ee Lui Ang

**Affiliations:** 1 Singapore Institute of Food and Biotechnology Innovation (SIFBI), Agency for Science, Technology, and Research (A*STAR), 31 Biopolis Way, #04-01, Nanos, Singapore, 138669 Republic of Singapore; 2 Department of Chemical and Biomolecular Engineering, University of Illinois at Urbana-Champaign, Urbana, IL 61801 USA; 3 Molecular Engineering Lab, Institute of Molecular and Cell Biology (IMCB), Agency for Science, Technology and Research (A*STAR), 61 Biopolis Drive, #07-06, Proteos, Singapore, 138673 Republic of Singapore; 4 Institute of Sustainability for Chemicals, Energy and Environment (ISCE2), Agency for Science, Technology and Research (A*STAR), 8 Biomedical Grove, #07-01 Neuros Building, Singapore, 138665 Republic of Singapore; 5 Synthetic Biology Translational Research Program, Yong Loo Lin School of Medicine, National University of Singapore, 10 Medical Drive, Singapore, 117597 Republic of Singapore

## Abstract

**Background:**

*Streptomyces* is renowned for its robust biosynthetic capacity in producing medically relevant natural products. However, the majority of natural products biosynthetic gene clusters (BGCs) either yield low amounts of natural products or remain cryptic under standard laboratory conditions. Various heterologous production hosts have been engineered to address these challenges, and yet the successful activation of BGCs has still been limited. In our search for a valuable addition to the heterologous host panel, we identified the strain *Streptomyces* sp. A4420, which exhibited rapid initial growth and a high metabolic capacity, prompting further exploration of its potential.

**Results:**

We engineered a polyketide-focused chassis strain based on *Streptomyces* sp. A4420 (CH strain) by deleting 9 native polyketide BGCs. The resulting metabolically simplified organism exhibited consistent sporulation and growth, surpassing the performance of most existing *Streptomyces* based chassis strains in standard liquid growth media. Four distinct polyketide BGCs were chosen and expressed in various heterologous hosts, including the *Streptomyces* sp. A4420 wild-type and CH strains, alongside *Streptomyces coelicolor* M1152, *Streptomyces lividans* TK24, *Streptomyces albus* J1074, and *Streptomyces venezuelae* NRRL B-65442. Remarkably, only the *Streptomyces* sp. A4420 CH strain demonstrated the capability to produce all metabolites under every condition outperforming its parental strain and other tested organisms. To enhance visualization and comparison of the tested strains, we developed a matrix-like analysis involving 15 parameters. This comprehensive analysis unequivocally illustrated the significant potential of the new strain to become a popular heterologous host.

**Conclusion:**

Our engineered *Streptomyces* sp. A4420 CH strain exhibits promising attributes for the heterologous expression of natural products with a focus on polyketides, offering an alternative choice in the arsenal of heterologous production strains. As genomics and cloning strategies progress, establishment of a diverse panel of heterologous production hosts will be crucial for expediting the discovery and production of medically relevant natural products derived from *Streptomyces*.

**Supplementary Information:**

The online version contains supplementary material available at 10.1186/s12934-024-02416-y.

## Background

The Actinobacteria phylum comprises high GC content microbes, featuring distinct fungal-like morphological development, sporulation, and unique biosynthetic capabilities [1]. Predominantly, natural product production, especially antibiotics, is centered in the *Streptomyces* genus [2, 3]. Their wide habitat range and interactions with diverse organisms led to the evolution of intricate natural product biosynthesis, captivating the drug discovery field [4]. Commercially, Actinobacteria have contributed an impressive array of natural product based drugs, including immunosuppressants like ascomycin and rapamycin [5, 6], antibiotics such as erythromycin and tetracycline [7, 8], and other compounds with applications spanning antifungal, antiviral, immunostimulant, anti-cancer, and agriculturally valuable properties [9, 10].

Native strains have complex regulatory systems, lack genetic manipulation tools and exhibit poor growth patterns, which result in ∼90% of biosynthetic gene clusters (BGCs) being cryptic under standard laboratory conditions or expressed at very low levels [11, 12]. However, recent breakthroughs in molecular biology and the development of innovative tools have redirected research interest toward the heterologous expression of these elusive BGCs in genetically well-understood and engineered chassis hosts [13–15]. Numerous *Streptomyces* strains have emerged as prime candidates for this purpose, with *Streptomyces coelicolor* leading the way as one of the most characterized and extensively studied species. Leveraging well-established molecular techniques and a deeply understood metabolism, *S. coelicolor* has proven invaluable in heterologous BGC expression [16]. In addition, closely related species like *Streptomyces lividans*, *Streptomyces avermitilis*, and *Streptomyces albus* have also played crucial roles in activating and enhancing the production of industrially relevant compounds [17–19]. These chosen heterologous hosts share common attributes, including rapid growth, abundant biosynthetic precursor availability, genetic manipulability, conjugation compatibility, and ideally, a low background of native metabolites [20]. However, the intricate genetic circuits and the complex nature of natural product biosynthesis necessitate the use of a diverse panel of heterologous hosts for expressing unknown BGCs [21, 22]. Consequently, there is an escalating demand for an array of *Streptomyces* heterologous hosts, as no single host alone can fulfill the ever-expanding requirements of this burgeoning field [23].

In the pursuit of enhancing the activation, discovery, and production of natural products, rigorous engineering efforts have been invested in various heterologous hosts [14]. Among these, the *S. coelicolor* M145 strain has undergone substantial modifications, initially involving the elimination of competing pathways responsible for actinorhodin, prodiginine, coelimycin, and calcium-dependent antibiotic production. This transformation yielded the "cleaner" background M1146 strain, facilitating the identification of heterologously expressed natural products. Subsequent iterations introduced previously identified advantageous *rpoB* (rifampicin resistance) or the double *rpoB* and *rpsL* mutations (streptomycin resistance) [24, 25], resulting in strains M1152 and M1154, respectively [26]. While these introduced mutations impacted growth, they led to remarkable increases in natural product yields, ranging from 20 to 40-fold.

Another closely related and extensively studied strain, *S. lividans* 66, garnered attention for its unique ability to accept methylated DNA and its low protease activity. Through the removal of self-replicating plasmids SLP2 and SLP3, the engineered strain TK24 emerged as one of the most widely adopted heterologous hosts [27]. Various modified *S. lividans* strains have displayed promising results in the production of the antibiotic daptomycin and the anti-cancer compound mithramycin A [28, 29]. Recently, a comprehensive re-evaluation of the *S. lividans* TK24 engineering concept was undertaken, involving the knockout of a total of nine metabolically active BGCs and the introduction of two additional *attB* integration sites to facilitate higher copy numbers of integrated heterologous BGCs [30]. The resultant strain, ΔYA11, exhibited superior production levels for three metabolites compared to its progenitor TK24 while maintaining robust growth performance, which outperformed even *S. coelicolor* M1152 strains.

Another chassis strain developed for natural products production was the minimized version of *S. albus* J1074 named Del14, where 15 native secondary metabolite biosynthetic pathways were deleted from the parental chromosome [31]. Interestingly, the introduction of additional *attB* integration sites led to only marginal improvements in production, while simultaneously compromising conjugation rates, casting doubts on the high BGC copy number strategy [32]. Both *S. albus* Del14 and *S. lividans* ΔYA11 strains displayed a proclivity for the expression of various BGCs sourced from the bacterial artificial chromosome (BAC) library of *Streptomyces albus* subsp. *chlorinus* NRRL B-24108. Overall, the highlighted studies demonstrated that the native competing pathways affected production of heterologous BGCs dramatically and selective knock-out strains improved final yields significantly. Furthermore, the reduction of background interference has not only streamlined the process but also improved the detection of heterologously expressed exogenous natural products.

In this study, we evaluate the prospectives of unique strains from an in-house collection as novel *Streptomyces* host for heterologous BGC production. The strain *Streptomyces* sp. A4420 was identified as part of the screening process for bacterial alkaloid producers, as the host for streptazolin natural product. Due to its innate affinity towards production of high levels of polyketides, which is one of the largest groups of natural products produced by bacteria, we decided to initially focus on evaluation of PKS-related gene clusters as even among polyketide class of natural products, there is a great diversity of scaffolds and post-PKS modifications to be investigated. To evaluate its capacity as a heterologous expression host, this strain was sequenced initially, followed by identification and deletion of 9 endogenous BGCs. The resulting engineered strain showed a similar growth pattern to that of the parental strain and outperformed commonly used *Streptomyces* heterologous expression hosts. Finally, by utilizing four heterologous Type I and II polyketide BGCs of varying chemical diversity as benchmarks including benzoisochromanequinone, glycosylated macrolide, glycosylated polyene macrolactam and heterodimeric aromatic polyketide products, a series of activation and production yield assessments were performed and juxtaposed against model organisms. These experiments demonstrate the feasibility and potential of our newly engineered strain to complement and significantly bolster the processes of natural product discovery and production. Collectively, our findings reveal that the newly engineered strain exhibits a range of promising features, positioning it as a valuable chassis strain to complement existing well-characterized heterologous hosts for natural product discovery and production.

## Results

### Strain identification and genetic analysis

**Fig. 1:**
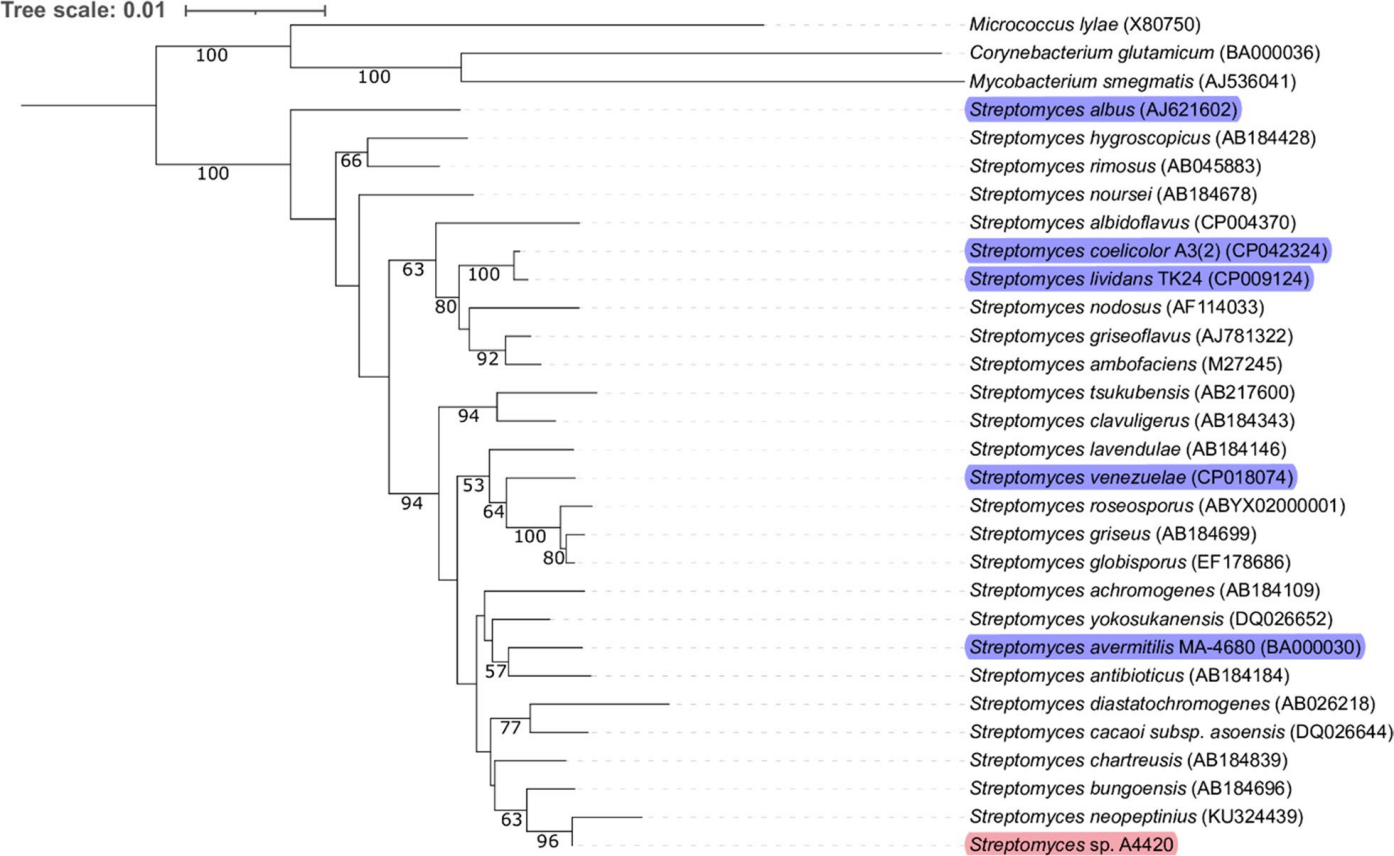
Neighbor-joining phylogenetic tree based on 16S rRNA gene sequences, showing the position of *Streptomyces* sp. A4420 relative to other well established heterologous hosts *S. lividans* TK24, *S. coelicolor* M1152, *S. albus* J1074 and *S. venezuelae* NRRL B-65442. Neighbor-joining tree shows distribution based on 243F and A3R amplified 16S rDNA samples from *Streptomyces* sp. A4420 strain. The root position was determined using *Micrococcus lylae*, *Corynebacterium glutamicum* and *Mycrobacterium smegmatis* as an outgroup. Bootstrap values are given as a percentage of 1000 replicates (only bootstrap values above 50% are shown). GenBank accession numbers are given in parentheses

*Streptomyces* sp. A4420 was identified from the private Natural Organism Library (NOL) collection housed within the Agency for Science and Technology (A*STAR) in Singapore [33]. Initial fermentation studies using solid SFM and ISP2 media showed high production of the piperidine alkaloid streptazolin reaching up to 10 mg L^−1^ yield, prompting us to investigate its potential as a chassis strain for natural products synthesis, in particular polyketides. At the same time, this strain also displayed a growth rate comparable to the commonly used *Streptomyces* heterologous host strains, and a uniquely high sporulation rate. To characterize its phylogenetic relationship to other *Streptomyces* strains, its 16S rDNA sequence was amplified using the established actinomycetes primer pair 243F and A3R [34] (Additional File 1: Fig. S1), and a phylogenetic tree comprising a total of 30 species was constructed using the MEGA 11 software with the neighbor-joining method [35, 36] (Fig. 1). This analysis revealed that *Streptomyces* sp. A4420 is distantly related to the *Streptomyces* strains commonly employed as heterologous hosts, including *S. albus* J1074, and the closely related *S. lividans* TK24 and *S. coelicolor* M1152. Among the strains previously used as heterologous hosts, *Streptomyces* sp. A4420 is most closely related to *Streptomyces avermitilis* MA-4680 [18], while among the sequenced strains, it is most closely related to *Streptomyces neopeptinius* strain F18.

### Construction of *Streptomyces* sp. A4420 CH strain for heterologous production of natural products

To obtain the genome sequence of the *Streptomyces* sp. A4420 strain, its genomic DNA (gDNA) was extracted, sequenced, and assembled following a recently described method for hybrid long-short read assembly of Illumina and Oxford Nanopore sequencing data [37]. This approach provides a cost-effective alternative to traditional whole genome sequencing techniques, enabling the investigation of a broader range of organisms as potential chassis strains. The assembled genome was analyzed using AntiSMASH [38] (Table 1), resulting in the identification of 9 Type I, II and NRPS hybrid polyketide BGCs, including the streptazolin BGC (Additional File 1: Fig. S2), which could potentially compete with exogenous BGCs for production. To improve precursor flux and eliminate production of undesired natural products in *Streptomyces* sp. A4420, these 9 polyketide BGCs were knocked out according to their identified boundaries. Due to concerns regarding Cas9-induced toxicity, potential off-target effects, and genome instability resulting from double stranded breaks [39–41], as well as lack of a comprehensive CRISPR-based genetic toolkit in this strain, we chose to employ traditional recombination-based techniques for genetic engineering.

**Table 1:** Summary of strains generated and corresponding BGCs knocked out

BGC	Type of BGC	Strain name	Deletion (kb)	Closest Homology
6	Type 1 PKS—NRPS	XW1	~ 72.8	Coelichelin
7	Type 1 PKS	XW2	~ 38.7	Geldanamycin
15	Type 1 PKS	XW3	~ 47.3	Maklamicin
22	Type 1 PKS	XW4	~ 76.9	Natamycin
31	Type 2 PKS	XW5	~ 47.5	Kinamycin
33	Type 2 PKS—NRPS	XW6	~ 62.7	Skyllamycin
39	Type 2 PKS	XW7	~ 37.7	Spore pigment
48	Type 1 PKS—NRPS	XW8	~ 70.1	Jawsamycin
42	Type 1 PKS	CH	~ 59.1	Streptazolin

Two homology arms flanking the target sequence were PCR-amplified from the purified gDNA of the wild-type *Streptomyces* sp. A4420 strain (WT) and cloned into a pIJ101 plasmid pYH7 [42, 43] that had been linearized with NdeI and HindIII by using HiFi assembly. The strain demonstrated high efficacy for intergeneric conjugation with *E. coli* ET12567 harboring plasmid pUZ8002, and this system was efficiently applied for the introduction of pYH7 plasmid. To identify mutants with double cross-over events, individual colonies were isolated for crude gDNA extractions, and successful recombination events were validated by PCR (Additional File 1: Table S2). This strategy was successfully employed to sequentially knock out all 9 target BGCs (Table 1), generating a chassis strain *Streptomyces* sp. A4420 CH (hereon annotated as CH strain). The engineered strain retained sensitivity to apramycin, indicating elimination of pYH7 suicide plasmid in the last round of mutations, allowing apramycin selection to be used for subsequent expression of exogenous BGCs.

### Biomass accumulation studies

**Fig. 2:**
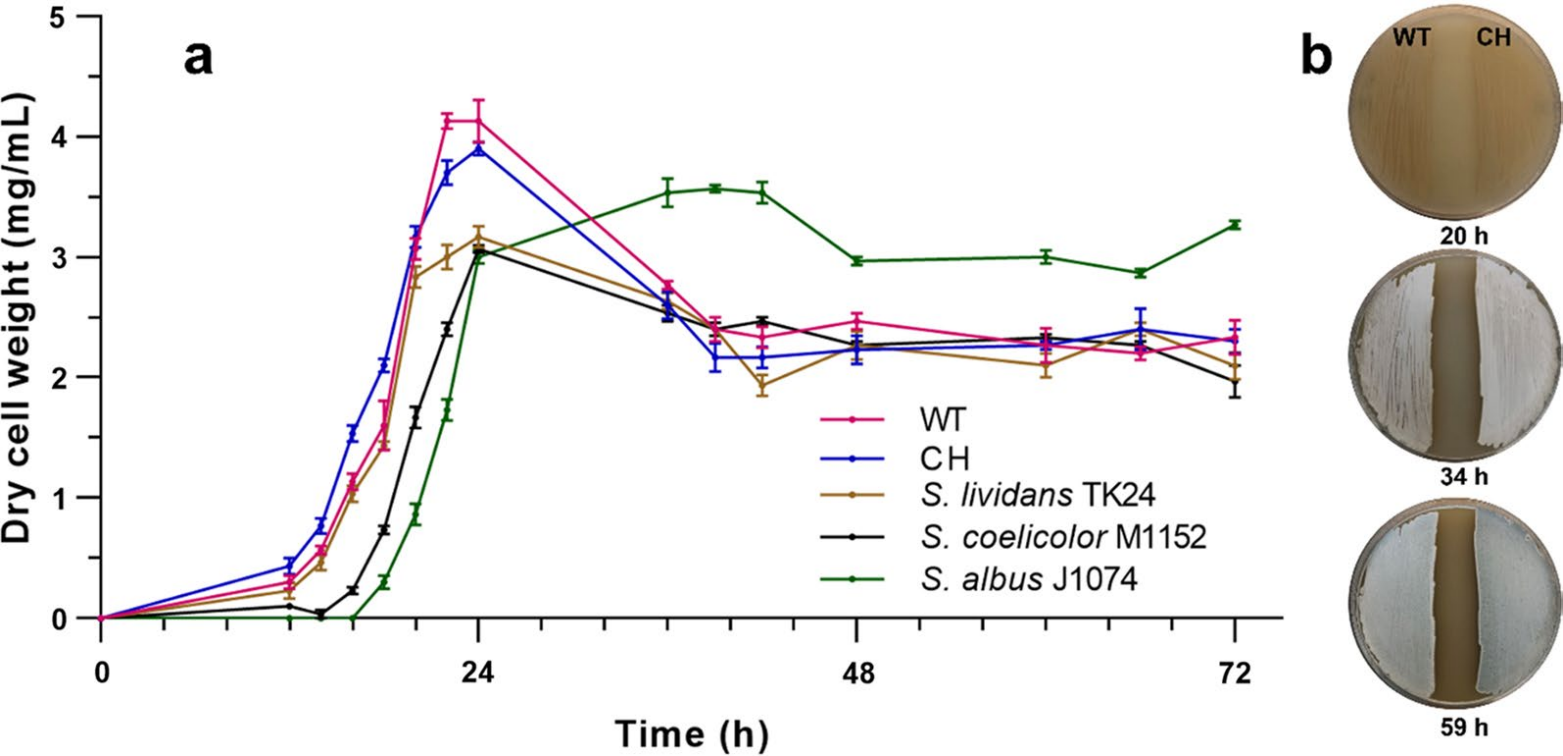
Biomass accumulation and morphological comparison studies. Growth curves for *Streptomyces* sp. A4420 (WT), *Streptomyces* sp. A4420 CH, *S. lividans* TK24, *S. coelicolor* M1152 and *S. albus* J1074 strains in TSB media supplemented with glass beads were established by plotting freeze dried cell pellet weight (**a**). Inoculation was normalized based on measured CFU of each spore stock. The *S. venezuelae* NRRL B-65442 strain was not used for cell growth experiments due to low observed sporulation. Solid phase growth of sporulation phenotype for *Streptomyces* sp. A4420 (WT) and *Streptomyces* sp. A4420 CH at was observed at 20, 34 and 59 h on SFM media (**b**)

Growth of the WT, CH, and three model chassis *Streptomyces* strains, *S. lividans* TK24, *S. coelicolor* M1152 and *S. albus* J1074, were compared in standard TSB media. Both WT and CH strains displayed a strong propensity for coagulation, therefore glass beads were added for agitation in all pre-culture and growth experiments. To accurately evaluate growth rates and minimize variations in initial density, inoculation was normalized based on colony forming unit (CFU), and dry biomass was quantified by freeze drying the collected samples. Under said conditions, both WT and CH displayed germination and growth rates similar to *S. lividans* TK24 strain and outperformed the other strains (Fig. 2a). In addition, WT and CH had the highest accumulated dry biomass, which peaked at 24 h before reaching a plateau at 39 h. The highest final biomass was observed for *S. albus* J1074, although it had the slowest germination and growth rate. No discernable differences in sporulation and growth were observed between WT and CH strains on SFM agar (Fig. 2b).

### Heterologous expression of exogenous BGCs

**Fig. 3:**
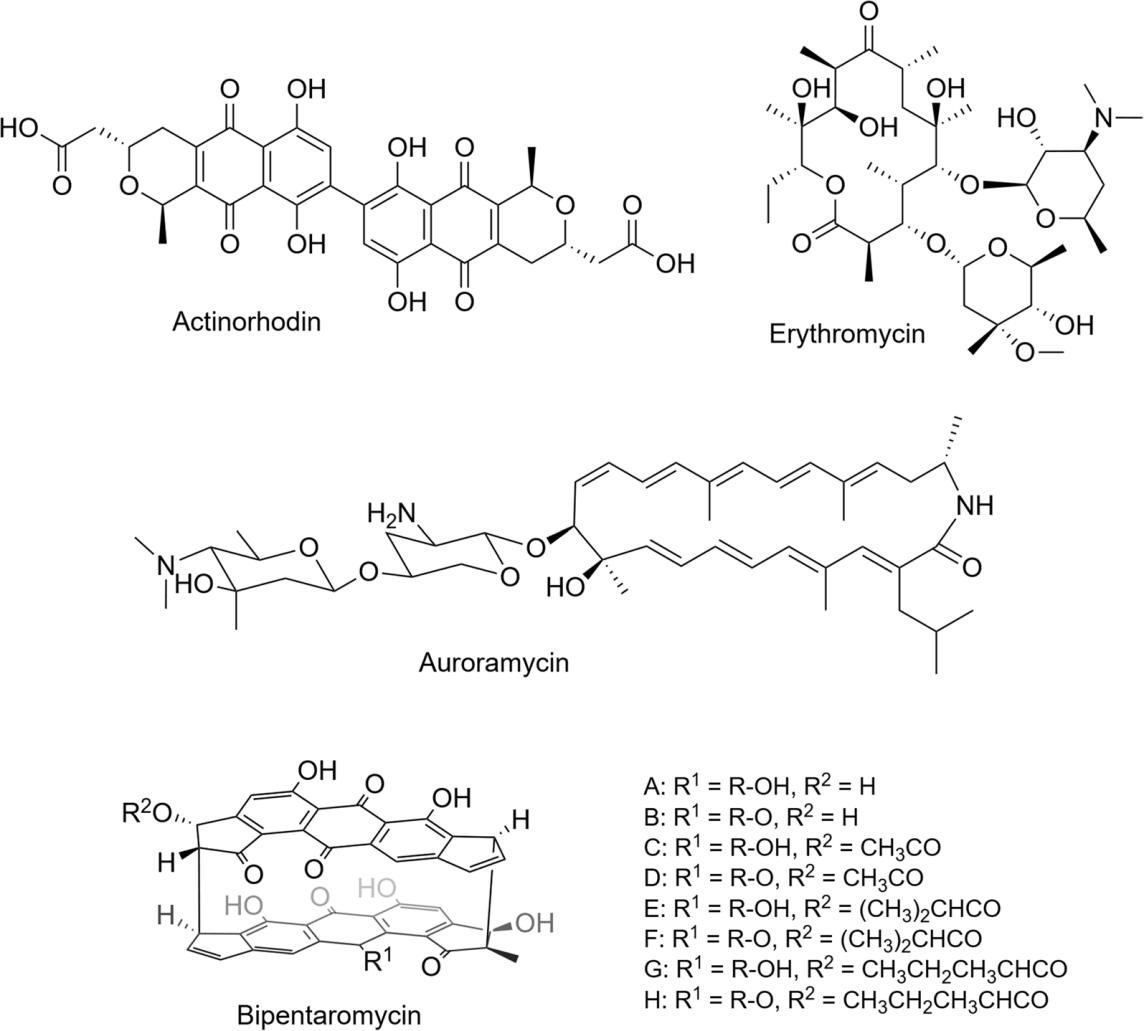
Structures of the natural products produced in the CH strain

**Fig. 4:**
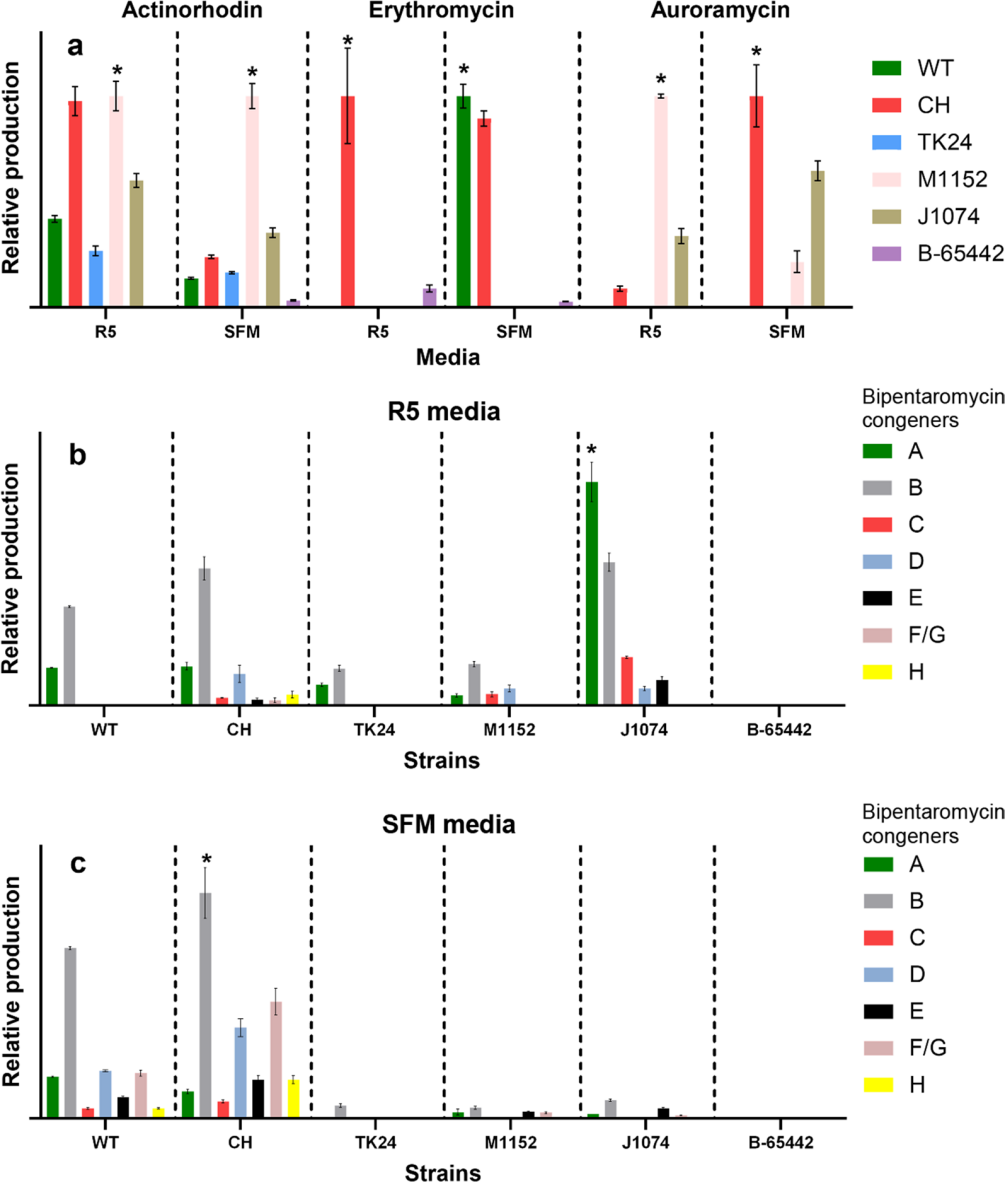
Heterologous production level of actinorhodin, erythromycin, and auroramycin (**a**), as well as bipentaromycin in R5 and SFM (**b,** and** c** respectively) in *Streptomyces* sp. A4420 (WT), *Streptomyces* sp. A4420 CH, *S. lividans* TK24, *S. coelicolor* M1152, *S. albus* J1074, *S. venezuelae* NRRL B-65442 in R5 and SFM media. Bipentaromycin congeners A–H as well as different strains for actinorhodin, erythromycin and auroramycin are highlighted in different colors. To facilitate comparison, the production scale is normalized to the highest producer depicted as 100% yield for each condition (highlighted with star on top). All production levels are represented with a linear scale, using A640 absorption (actinorhodin), peak area in BPC + mode (erythromycin and auroramycin) and peak area using A280 absorption (bipentaromycin). Expanded version is available at Additional File 1: Fig. S18

To evaluate the biosynthetic capacity of the CH strain, we selected four heterologous polyketide BGCs producing antibiotic and anti-fungal polyketides, including the well-studied actinorhodin [44] and erythromycin [45], together with the recently discovered auroramycin [46, 47] and bipentaromycin [48] (Fig. 3). The gene clusters were introduced into WT, CH, *S. lividans* TK24 [49], *S. coelicolor* M1152 [26], *S. albus* J1074 [50], and *S. venezuelae* NRRL B-65442 [51] via intergeneric conjugation using *E. coli* ET12567 containing a pUZ8002 plasmid donor. The auroramycin BGC, which has not previously been heterologously expressed, was cloned from *S. roseosporus* NRRL 15998 using the CAPTURE method [22] for this study. All BGCs were successfully conjugated with different efficiencies, as confirmed by PCR using internal primers (data not shown). Isolated exconjugants were inoculated into TSB media for pre-culture, followed by transfer into SFM and R5 fermentation media for a growth period of 7 days. Actinorhodin production rates were directly evaluated using a colorimetric assay [52], while other fermentation samples were freeze dried, extracted with methanol, and analysed by LC–MS. In general, production of the compounds by different strains varied between the two media. The CH strain produced all four compounds in both media (Fig. 4) (Additional File 1: Fig. S3–S15), and significantly outperformed the parental strain under all conditions, except for erythromycin in SFM media where both strains showed similar production levels.

For actinorhodin, *S. coelicolor* M1152 showed the highest production in SFM media, with M1152 and the CH strain being matched for the highest production in R5 media (Fig. 4a). The WT strain showed only 50% of the production levels of the CH strain in both media. Erythromycin production was detected for CH in both media, and a similar production level was detected for WT in SFM, but not in R5 media. Interestingly, no erythromycin production was detected in either of *S. lividans* TK24, *S. coelicolor* M1152, *S. albus* J1074 in both media, except for low levels in *S. venezuelae* NRRL B-65442 (Fig. 4a). Auroramycin production was observed for the CH strain, *S. coelicolor* M1152 and *S. albus* J1074 at different levels in both media (Fig. 4a), while only trace levels were observed for WT, *S. lividans* TK24 and *S. venezuelae* NRRL B-65442 in R5 media. Bipentaromycins are heterodimeric aromatic molecules comprising two distinctive pentacyclic ring systems, with two specific modification sites R^1^ and R^2^, allowing us to investigate the ability of the various strains to produce a range of congeners (A-H in Figs. 3, 4b, c). In R5 media, the highest production level was observed for *S. albus* J1074, followed by the CH and WT strains, with much lower or undetectable levels for the other strains. In SFM media, production of all seven congeners was observed for the CH and WT strains, with much lower or undetectable levels for the other strains.

**Fig. 5:**
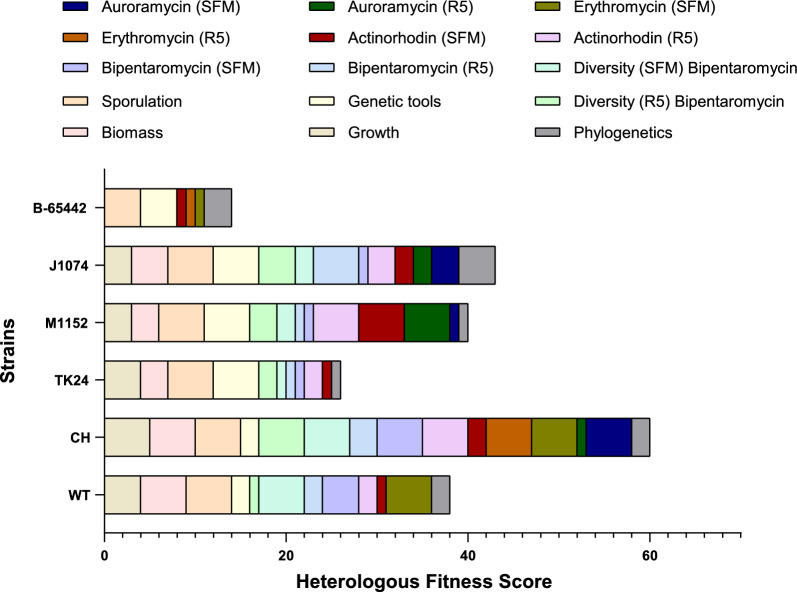
Multi parameter evaluation and comparison of heterologous fitness score of *Streptomyces* sp. A4420 (WT), *Streptomyces* sp. A4420 CH, *S. lividans* TK24, *S. coelicolor* M1152, *S. albus* J1074, *S. venezuelae* NRRL B-65442 based on determined parameters (Additional File 1: Table S4)

Consequently, we proceeded to assess the performance and suitability of our engineered CH strain as a candidate for heterologous production based on 15 parameters, which encompass data from this study and previously published sources (Additional File 1: Table S4, Fig. S18 and S19). We introduced a scoring system to rank the performance of each strain across these individual parameters. To mitigate bias towards any single criterion, we summed the performance scores to create a heterologous fitness score. This allowed for a direct comparison of all the strains tested under the specific conditions in this study (Fig. 5). *S. venezuelae* NRRL B-65442, which is not commonly used as a heterologous host, exhibited a notably lower heterologous fitness score compared to the other strains. Surprisingly, *S. lividans* TK24, one of the commonly explored heterologous producers, scored lower than other strains. This outcome may be attributed to the specific nature of the BGCs that were tested.

## Discussion

Actinobacteria offer a rich source of natural products for medical applications, and research into their secondary metabolism has contributed immensely to drug discovery. Their genomes host a multitude of BGCs, most of which remain dormant under standard lab conditions, suggesting that their full biosynthetic capabilities remain underexplored [3, 53]. Advances in DNA sequencing have expanded our knowledge of their native regulatory circuits and BGC organization, setting the stage for the exploration of new BGCs through heterologous expression. *Streptomyces* strains like *S. coelicolor*, *S. lividans*, *S. albus*, and *S. avermitilis* have been instrumental in discovering natural products, and in advancing our knowledge of their genetics, biochemistry, and physiology [19]. The recently developed CAPTURE direct cloning strategy enables large-scale cloning of BGCs from various *Streptomyces* strains, and has revealed the limited capacity of widely used heterologous hosts to functionally express exogenous gene clusters [22], with only approximately 16% of the studied natural product pathways being successfully expressed.

To broaden the selection of *Streptomyces* strains available as heterologous hosts, we investigated *Streptomyces* sp. A4420, which is a prolific producer of streptazolin, suggesting a strong potential for production of other polyketides as well. A 16S rRNA analysis revealed substantial divergence from well-characterized *Streptomyces* strains, prompting a comprehensive assessment of its genomics and metabolomics. To obtain the genomic sequence of the strain for engineering, we explored the cost-effective combination of Illumina and Oxford Nanopore sequencing [37]. AntiSMASH analysis [38] resulted in identification of the streptazolin cluster and other potentially competing polyketide BGCs, facilitating the construction of a *Streptomyces* sp. A4420-based CH strain by sequential knockouts of 9 annotated and potentially competing polyketide BGCs. Alternatively, comprehensive understanding native secondary metabolite biosynthetic pathways as well as of genetic elements responsible for growth cycle, genetic stability, intracellular energy dynamics and redox potential can be effectively used to pre-select and rationally design large deletions as demonstrated for *Streptomyces chattanoogensis* L10 [54]. However, determining core conserved regions as well as dispensable sub-telomeric areas is time consuming and was predominantly explored in model strains only [55]. The metabolically streamlined chassis strain exhibited no changes in sporulation or growth, and yielded comparable biomass in liquid culture.

To assess the potential of our engineered CH strain as a novel heterologous producer, we selected four diverse polyketides, including actinorhodin (benzoisochromanequinone, produced by a type II PKS), erythromycin (glycosylated, macrolide, produced by a type I PKS), auroramycin (glycosylated, polyene macrolactam, produced by a type I PKS) and bipentaromycin (heterodimeric aromatic polyketide, produced by a type II PKS). The engineered CH strain and parental strain were compared against well-established heterologous hosts such as *S. coelicolor* M1152, *S. lividans* TK24, *S. albus* J1074, and the model strain *S. venezuelae* NRRL B-65442 commonly used in morphological studies (Fig. 4). Variable conjugation rates were observed depending on the tested BGC, with bipentaromycin displaying the lowest efficiency, resulting in fewer than 10 exconjugants for each strain. The deletion of endogenous competing BGCs led to enhanced natural product production in the CH strain compared to the parental strain in all cases, except for a single condition involving the erythromycin BGC. This suggests that complete genome minimization may not be imperative for the development of a heterologous producer, at least in the context of improving the yields of the investigated compounds. Simultaneously, glycosylated polyketides like erythromycin and auroramycin were not detected in the parental *Streptomyces* sp. A4420 (except for erythromycin in SFM medium) but were successfully reconstituted in the metabolically simplified strain when cultured in R5 and SFM media, respectively.

Interestingly, erythromycin was undetectable in any of the model heterologous secondary metabolite expression strains, including *S. lividans* TK24, *S. coelicolor* M1152, and *S. albus* J1074, which led us to suspect that part of the BGC might have been lost due to recombination events. However, PCR analysis using five pairs of primers, annealing at different regions of the BGC, indicated that the cluster remained intact (Additional File 1: Figs. S16, S17). Indeed, heterologous expression of glycosylated erythromycin could not be reconstituted in *S. coelicolor* and *S. lividans* production hosts, only yielding intermediate metabolites [56]. Simultaneously, *S. venezuelae* NRRL B-65442 showed detectable levels of erythromycin, emphasizing that the issue likely stems from intrinsic regulatory circuits. It is worth noting that both *S. lividans* TK24 and *S. coelicolor* M1152 share many metabolic pathways and potentially regulatory circuits due to their close genetic relatedness [27]. This conclusion is supported by our 16S rRNA analysis, which revealed their similar phylogenetic niche. It further underscores the necessity for distinctively diverse heterologous hosts with a supply of unusual precursors and diverse regulatory systems.

*S. coelicolor* M1152 is a metabolically streamlined derivative of the strain M145. The parental strain is known for being a prolific producer of the pigmented antibiotic actinorhodin, and the derivative strain is equally prolific when the actinorhodin gene cluster in re-introduced [26, 57], while the closely related *S. lividans* strain demonstrates only moderate yields. This observation suggests that these strains possess well-established precursor flux, regulatory, and secretion systems for antibiotic resistance [52]. Thus, we anticipated that our engineered CH strain might encounter challenges with this particular gene cluster. Indeed, in SFM media, *S. coelicolor* M1152 exhibited significantly higher production levels. However, to our surprise, the production rates in R5 media were comparable between the CH and the latter *S. coelicolor* M1152 strain. To push the boundaries further, we selected the cryptic auroramycin BGC, spanning approximately 106 kb, from *S. roseosporus* NRRL 15 998 [46, 58]. Using our recently described CAPTURE method [22] and from our in-house strain library, we cloned and expressed this BGC heterologously. This approach enabled the decoupling of native regulatory elements, and the glycosylated polyketide was detectable in three of the tested strains, though at varying production levels. In this instance, the removal of competing BGCs from the parental *Streptomyces* sp. A4420 was indispensable for detecting and producing auroramycin, albeit at relatively low levels. The most structurally diverse BGC investigated in this study, bipentaromycin, could be effectively expressed only in *S. avermitilis* SUKA 17, as reported previously [48]. Both the parental and metabolically simplified CH strains demonstrated the capability to produce all main derivatives A to H at detectable levels compared to the other tested strains. Interestingly, the limitations in this case did not stem from a lack of available precursors but rather from the heterologous expression of modifying enzymes, such as Bpa9 and Bpa15, which give rise to hydroxylated and methylated derivatives. BGC arrangements are intricate, encompassing not only core biosynthetic genes but also regulatory circuits, transporters, and post-tailoring enzymes [59].

These findings affirm that, under specified conditions, each of the strains can serve as a highly efficient host for heterologous production of natural products. Our analysis suggests that simplifying the secondary metabolite profile of *Streptomyces* sp. A4420 significantly improved the heterologous fitness score (Fig. 5), surpassing even the performance of *S. coelicolor* M1152 and *S. albus* J1074 strains. While this analysis can be subject to the definition and criteria of the scoring system, it represents our initial attempt to develop a more comprehensive approach to visualize the impact of extensive strain selection and engineering. In the future, we aim to expand the range of tested conditions and refine our comparison strategy. This expansion should encompass recently engineered and validated strains such as *S. lividans* TK24 ΔYA11 [30], *S. albus* J1074 Del14 [31], and *S. avermitilis* SUKA strains [18]. Additionally, a broader array of BGCs and different culture media could be included to provide a more comprehensive performance evaluation.

## Conclusion

In summary, we have discovered and engineered an alternative CH chassis strain derived from *Streptomyces* sp. A4420 for natural product biosynthesis. Both the engineered and parental strains exhibited faster growth rates and higher accumulated biomass than other commonly used *Streptomyces* strains. Compared to the parental strain, the engineered strain exhibited improved heterologous production of polyketide natural products, with yields on par with or surpassing those of commonly used strains under specific conditions. To facilitate a comprehensive comparison, we introduce a heterologous fitness score, which demonstrates that the engineered CH strain outperforms the parental strain under all evaluated criteria, and also compares favorably to commonly used strains, including *S. albus* J1074*, S. coelicolor* M1152, and *S. lividans* TK24. The favorable growth and production characteristics of this metabolically streamlined *Streptomyces* strain make it an attractive chassis strain for the discovery and production of polyketides, while further assessments are underway for other classes of natural products. In-depth genomic, transcriptional and translational analysis of the CH strain and the associated BGCs will aid in understanding the basis of its biosynthetic capacity for polyketides. This will also guide the continued genomic optimization of the CH strain, and development of its genome engineering tools and strategies, to enable its integration into the repertoire of commonly employed heterologous hosts.

## Methods

### Strains, plasmids, and culturing conditions

All the *E. coli* and *Streptomyces* strains, and plasmids used in this study are provided in Additional File 1: Table S1. *Streptomyces* sp. A4420 was obtained from private Natural Organism Library (NOL) collection housed within the Agency for Science and Technology (A*STAR, Singapore). *S. coelicolor* M1152, *S. albus* J1074 and *S. venezuelae* NRRL B-65442 were kindly provided by Jason Micklefield (University of Manchester, UK). NEB10β cell line was used for propagation of plasmids encoding heterologous BGC and *E. coli* ET12567 harboring pUZ8002 plasmid, for intergeneric conjugation with *Streptomyces* strains. *E. coli* strains were cultivated in LB (lysogeny broth) and 2xYT (Sigma-Aldrich, USA) for general cloning and conjugation respectively. *Streptomyces* strains were grown on soy flour mannitol (SFM) solid medium for sporulation (20 g soya flour, 20 g D-mannitol, 20 g agar, 1 L ddH_2_O) or tryptic soy broth (TSB) for pre-culture (Sigma-Aldrich, USA). Soy flour mannitol (MS) (20 g soya flour, 20 g D-mannitol, 1 L ddH_2_O) and R5 liquid medium [27] were used for heterologous BGC expression and natural product production. *E. coli* and *Streptomyces* strains were cultured at 37 °C and 30 °C respectively with 250 rpm agitation for liquid cultures. Antibiotics were used as following: apramycin (50 µg/mL), thiostrepton (25 µg/mL) and chloramphenicol (25 µg/mL).

### Genomic DNA isolation, manipulation, and sequencing

Genomic DNA from *Streptomyces* was isolated using lysozyme-based lysis with phenol/chloroform extraction method. Full genome sequence was obtained according to recently described Illumina and Oxford Nanopore hybrid sequencing strategy [37]. Intergeneric conjugation between *E.* coli ET12567/pUZ8002 and *Streptomyces* strains was performed according to previously established protocols [27]. PrimeSTAR Max (Takara Bio, Japan) and GoTaq® Green (Promega, USA) polymerases were used for cloning and PCR verification, respectively. The primers used in the study are listed in Additional File 1: Table S3. PCR-amplified fragments were analyzed and purified from agarose gels using the QIAquick Gel Extraction Kit (Qiagen, Germany). All restriction enzymes and NEBuilder HiFi DNA assembly mastermix were used according to manufacturer’s recommendations (New England Biolabs, USA).

### Biomass accumulation studies

Biomass accumulation was measured for all strains (except *S. venezuelae* NRRL B-65442) at 30 °C and 250 rpm agitation in 500 mL baffled flasks. 66 × 10^6^ CFU of each spore stock was used to inoculate 100 mL TSB growth medium supplemented with 55 g, 5 mm glass beads (Sigma-Aldrich, USA). 1 mL of each culture was collected at respective time points in pre-weighted Eppendorf tubes and centrifuged at 5000×*g* for 10 min at 4 °C. Supernatant was discarded and cell pellets were washed twice with equal amount of ddH_2_O. Wet cell pellets were freeze-dried overnight, and dry biomass weight was measured using Ohaus PA 214C Pioneer Series Analytical Balance.

### Construction of *Streptomyces* sp. A4420 CH strain for heterologous production of natural products

To generate a strain with desired genotype, sequential knock out steps were performed iteratively using pYH7 suicide plasmid and homologous recombination [42, 43] removing entire BGCs based on AntiSMASH prediction of the cluster boundaries. *Streptomyces*—*E. coli* shuttle vector pYH7 was digested with NdeI and HindIII followed by HiFi cloning with the two flanking homology arms amplified by PCR to generate pYH7_XW1 (Additional File 1: Table S1). The verified construct was transformed to *E. coli* ET12567/pUZ8002 strain followed by conjugation with *Streptomyces* sp. A4420 strain. Thiostrepton and apramycin resistant exconjugants were initially selected on SFM media supplemented with 10 mM MgCl_2_. After sporulation double cross-over mutants were selected based on apramycin and thiostrepton sensitivity, yielding XW1 strain (Additional File 1: Table S2). The genotype of mutants was confirmed using PCR analysis of amplified bands. Knock out process was repeated with the latter strain and subsequently generated strains until the last mutant was acquired with the genotype containing all selected mutations.

### Heterologous production and extraction of natural products

Spores harboring heterologous BGCs were used to inoculate 30 mL TSB supplemented with 12.5 mL 5 mm glass beads. The pre-culture was grown for 24–36 h at 30 °C, 250 rpm until cloudy culture observed, and 50 mg of wet biomass used to inoculate 25 mL fermentation media in triplicates. Strains were incubated for 7 days at 30 °C and 250 rpm agitation. The metabolites were extracted as following: actinorhodin fermentation samples were mixed with 25 mL 1 M KOH (1:1 ratio) according to previously established protocol [52]. For erythromycin, bipentaromycin and auroramycin the whole culture was freeze-dried until all moisture was removed. 35 mL of MeOH was added to dry cultures and extracted using sonication bath at room temperature for 30 min. Samples then vortexed for 30 s and soluble supernatant directly injected into LCMS.

### Analysis and evaluation of natural products

Actinorhodin samples were measured directly at 640 nm after addition of 1 M KOH. 10 µL of erythromycin, bipentaromycin and auroramycin extracts were separated by using Thermo Scientific Ultimate3000 RS system equipped with a Phenomenex Kinetex 2.6 µm XB-C18 100 Å (150 mm × 4.6 mm) and running the following program: acetonitrile + 0.1% formic acid against water + 0.1% formic acid from 5 to 50% (0–15 min), 50 to 100% (15–20 min), 100% (isocratic elution, 20–24 min), 100 to 5% (24–25 min), 5% (isocratic elution, 25–30 min) at 0.6 mL/min flow rate. Eluents were analyzed using Bruker amaZon SL mass spectrophotometer system. Mass spectra were acquired in centroid mode ranging from 100 to 2000 m*/z* at a 2 Hz scan rate. Production of target natural products and yields were evaluated as following: erythromycin peak was identified based on the acquired standard and relative yields were compared using BPC + mode; bipentaromycin peaks were identified based on previously determined m/z data [48] and yields were compared using A280 absorption; auroramycin peak was identified using a combination of native activated producer and m/z data published [46], and relative yields were evaluated using BPC + mode.

### Supplementary Information

**Additional file 1.** Supplementary tables and figures.

## Data Availability

The datasets used and/or analysed during the current study are available from the corresponding author on reasonable request.
