# Supplementary material for: Discovery, characterization, and engineering of an advantageous Streptomyces host for heterologous expression of natural product biosynthetic gene clusters
Source: Microb Cell Fact. 2024 May 24;23:149. doi: 10.1186/s12934-024-02416-y (PMC11127301; doi:10.1186/s12934-024-02416-y)
Supplement: Supplementary file 1 — Additional file 1. Supplementary tables and figures. [file 12934_2024_2416_MOESM1_ESM.docx]

**Additional File 1 – Supplementary Information**

**Discovery, characterization, and engineering of an advantageous *Streptomyces* host for heterologous expression of natural product biosynthetic gene clusters**

Evaldas Klumbys^1^, Wei Xu^1^, Lokanand Koduru^3^, Elena Heng^3^, Yifeng Wei^1^, Fong Tian Wong^3,4^, Huimin Zhao^1, 2, *^ and Ee Lui Ang^1,4,5*^

^1^ Singapore Institute of Food and Biotechnology Innovation (SIFBI), Agency for Science, Technology, and Research (A*STAR), 31 Biopolis Way, #04-01, Nanos, Singapore 138669, Republic of Singapore.

^2^ Department of Chemical and Biomolecular Engineering, University of Illinois at Urbana-Champaign, Urbana, IL 61801

^3^ Molecular Engineering Lab, Institute of Molecular and Cell Biology (IMCB), Agency for Science, Technology and Research (A*STAR), 61 Biopolis Drive, #07-06, Proteos, Singapore 138673, Republic of Singapore.

^4^ Institute of Sustainability for Chemicals, Energy and Environment (ISCE^2^), Agency for Science, Technology and Research (A*STAR), 8 Biomedical Grove, #07-01 Neuros Building, Singapore 138665, Republic of Singapore.

^5^ Synthetic Biology Translational Research Program, Yong Loo Lin School of Medicine, National University of Singapore, 10 Medical Drive, Singapore 117597, Republic of Singapore.

*To whom correspondence should be addressed.

Huimin Zhao: Phone: (217) 333-2631. E-mail: [zhao5@illinois.edu](mailto:zhao5@illinois.edu)

Ee Lui Ang: Phone: (65) 6279 2725. Email: ang_ee_lui@sifbi.a-star.edu.sg

**Table S1**. Strains and plasmids used in this study.

| **Strain** | **Characteristic** | **Reference** |
| --- | --- | --- |
| ***Escherichia coli*** | | |
| **NEB10β** | CAPTURE cloning | [1] |
| **ET12567/pUZ8002** | Donor strain for conjugation  between *E. coli* and *Streptomyces* hosts | [2] |
| ***Streptomyces* strains** | | |
| **A4420** | Streptazolin producing wild type strain (*Streptomyces* sp. A4420) | This study |
| ***S. lividans* TK24** | Wild type *S. lividans* strain without native plasmid | [3] |
| ***S. coelicolor* M1152** | Derivative of *S. coelicolor* A(3)2 strain | [4] |
| ***S. venezuelae* NRRL B-65442** | Wild type *S. venezuelae* strain | [5] |
| ***S. albus* J1074** | Wild type *S. albus* strain | [6] |
| ***S. roseosporus* NRRL 15998** | *Streptomyces* strain with cryptic auroramycin BGC | [7] |
| ***S. roseosporus* NRRL 15998 Rose22** | Auroramycin producing activated *S. roseosporus* NRRL 15 998 containing 97 bp *kasO** promoter | [7] |
| **Knock out strains** | | |
| **XW1** | A4420 containing **Δ006** (**Deletion of type I PKS – NRPS gene)** | This study |
| **XW2** | **XW1** containing additional **Δ007** (**Deletion of type I PKS gene)** | This study |
| **XW3** | **XW2** containing additional **Δ015** (**Deletion of type I PKS gene)** | This study |
| **XW4** | **XW3** containing additional **Δ022** (**Deletion of type I PKS gene)** | This study |
| **XW5** | **XW4** containing additional **Δ031 (Deletion of type II PKS gene)** | This study |
| **XW6** | **XW5** containing additional **Δ033(Deletion of type II PKS – NRPS gene)** | This study |
| **XW7** | **XW6** containing additional **Δ039(Deletion of type II PKS gene)** | This study |
| **XW8** | **XW7** containing additional **Δ048** (**Deletion of type I PKS – NRPS gene)** | This study |
| **XW9 – CH** | **XW8** containing additional **Δstl (Deletion of streptazolin gene)**  Δ006Δ007Δ015Δ022Δ031Δ033Δ039Δ048Δstl final genotype. Metabolically simplified *Streptomyces* sp. A4420 based chassis strain | This study |
| ***Streptomyces* strains for heterologous BGC expression** | | |
| **A4420::Act** | **Wild type** *Streptomyces* sp. A4420 strain with heterologous actinorhodin BGC | This study |
| **A4420::Ery** | **Wild type** *Streptomyces* sp. A4420 strain with heterologous erythromycin BGC | This study |
| **A4420::Bipent** | **Wild type** *Streptomyces* sp. A4420 strain with heterologous bipentaromycin BGC | This study |
| **A4420::Aur** | **Wild type** *Streptomyces* sp. A4420 strain with heterologous auroramycin BGC | This study |
| **CH::Act** | Metabolically simplified *Streptomyces* sp. A4420-CH strain with heterologous actinorhodin BGC | This study |
| **CH::Ery** | Metabolically simplified *Streptomyces* sp. A4420-CH strain with heterologous erythromycin BGC | This study |
| **CH::Bipent** | Metabolically simplified *Streptomyces* sp. A4420-CH strain with heterologous bipentaromycin BGC | This study |
| **CH::Aur** | Metabolically simplified *Streptomyces* sp. A4420-CH strain with heterologous auroramycin BGC | This study |
| **1074::Act** | ***S. albus* J1074** with heterologous actinorhodin BGC | This study |
| **1074::Ery** | ***S. albus* J1074** with heterologous erythromycin BGC | This study |
| **1074::Bipent** | ***S. albus* J1074** with heterologous bipentaromycin BGC | This study |
| **1074::Aur** | ***S. albus* J1074** with heterologous auroramycin BGC | This study |
| **TK24::Act** | ***S. lividans* TK24** with heterologous actinorhodin BGC | This study |
| **TK24::Ery** | ***S. lividans* TK24** with heterologous erythromycin BGC | This study |
| **TK24::Bipent** | ***S. lividans* TK24** with heterologous bipentaromycin BGC | This study |
| **TK24::Aur** | ***S. lividans* TK24** with heterologous auroramycin BGC | This study |
| **M1152::Act** | ***S. coelicolor* M1152** with heterologous actinorhodin BGC | This study |
| **M1152::Ery** | ***S. coelicolor* M1152** with heterologous erythromycin BGC | This study |
| **M1152::Bipent** | ***S. coelicolor* M1152** with heterologous bipentaromycin BGC | This study |
| **M1152::Aur** | ***S. coelicolor* M1152** with heterologous auroramycin BGC | This study |
| **Ven::Act** | ***S. venezuelae* NRRL B-65442** with heterologous actinorhodin BGC | This study |
| **Ven::Ery** | ***S. venezuelae* NRRL B-65442** with heterologous erythromycin BGC | This study |
| **Ven::Bipent** | ***S. venezuelae* NRRL B-65442** with heterologous bipentaromycin BGC | This study |
| **Ven::Aur** | ***S. venezuelae* NRRL B-65442** with heterologous auroramycin BGC | This study |
|  | | |
| **Plasmids** | **Characteristic** | **Reference** |
| pYH7 | *E. coli* and *Streptomyces* shuttle vector acting as a self-replicating vector | [8] |
| pBE14 | CAPTURE cloning plasmid | [1] |
| pBE45 | CAPTURE cloning plasmid | [1] |
| pBE48 | CAPTURE cloning plasmid | [1] |
| pBE-Act | Actinorhodin BGC containing shuttle vector | [1] |
| pBE-Ery | Erythromycin BGC containing shuttle vector | [1] |
| pBE-Bipent | Bipentaromycin BGC containing shuttle vector | [1] |
| pBE-Aur | Auroramycin BGC containing shuttle vector | This study |
| **Knock out plasmids** | | |
| **pYH7_XW1** | pYH7 shuttle vector containing 2 homology arms for knocking out type I PKS – NRPS hybrid (BGC006). pYH7 plasmid treated with NdeI and HindIII for HiFi cloning with PCR amplified Left and Right homology arms from A4420 strain. | This study |
| **pYH7_XW2** | pYH7 shuttle vector containing 2 homology arms for knocking out type I PKS (BGC007). pYH7 plasmid treated with NdeI and HindIII for HiFi cloning with PCR amplified Left and Right homology arms from A4420 strain. | This study |
| **pYH7_XW3** | pYH7 shuttle vector containing 2 homology arms for knocking out type I PKS (BGC015). pYH7 plasmid treated with NdeI and HindIII for HiFi cloning with PCR amplified Left and Right homology arms from A4420 strain. | This study |
| **pYH7_XW4** | pYH7 shuttle vector containing 2 homology arms for knocking out type II PKS (BGC022). pYH7 plasmid treated with NdeI and HindIII for HiFi cloning with PCR amplified Left and Right homology arms from A4420 strain. | This study |
| **pYH7_XW5** | pYH7 shuttle vector containing 2 homology arms for knocking out type II PKS – NRPS (BGC031). pYH7 plasmid treated with NdeI and HindIII for HiFi cloning with PCR amplified Left and Right homology arms from A4420 strain. | This study |
| **pYH7_XW6** | pYH7 shuttle vector containing 2 homology arms for knocking out type II PKS (BGC033). pYH7 plasmid treated with NdeI and HindIII for HiFi cloning with PCR amplified Left and Right homology arms from A4420 strain. | This study |
| **pYH7_XW7** | pYH7 shuttle vector containing 2 homology arms for knocking out type II PKS (BGC039). pYH7 plasmid treated with NdeI and HindIII for HiFi cloning with PCR amplified Left and Right homology arms from A4420 strain. | This study |
| **pYH7_XW8** | pYH7 shuttle vector containing 2 homology arms for knocking out type I PKS – NRPS (BGC048). pYH7 plasmid treated with NdeI and HindIII for HiFi cloning with PCR amplified Left and Right homology arms from A4420 strain. | This study |
| **pYH7_XW9** | pYH7 shuttle vector containing 2 homology arms for knocking out type I PKS (BGC042 – streptazolin). pYH7 plasmid treated with NdeI and HindIII for HiFi cloning with PCR amplified Left and Right homology arms from A4420 strain. | This study |
|  |  |  |

**Table S2**. PCR verification of generated sequential knock out strains starting from the wild type A4420 strain. Each of the following gels are arranged in order of sequential knock out mutations introduced. Ladder – 1^st^ base 1 kb DNA ladder, WT – wild type A4420 strain gDNA used as a negative control, pYH7_XW1 – pYH7_XW7 – pYH7 shuttle vector containing 2 homology arms (based on Table S1). ET12567/pUZ8002 *E. coli* donor strain was used for intergeneric conjugation with knock out plasmids. Each sequential mutant was screened using a specific pair of primers and bands compared against the wild type strain and knock out plasmid. Successful mutants were selected for the next round of mutations. Green arrow indicates expected band size for successful knock out event and the number suggest expected size.

| **Knock out verification** | **Knock out description** |
| --- | --- |
| 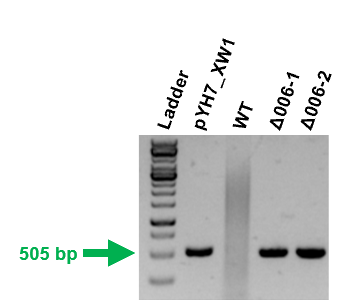 | Knock out of wild type A4420 strain BGC006 using pYH7_XW1 plasmid generating XW1 strain (Δ006). Primers used for homology arms: pYH7_XW1_L_F/pYH7_XW1_L_R and pYH7_XW1_R_F/pYH7_XW1_R_F. Validated using primers: XW1_CK_F/XW1_CK_R |
| 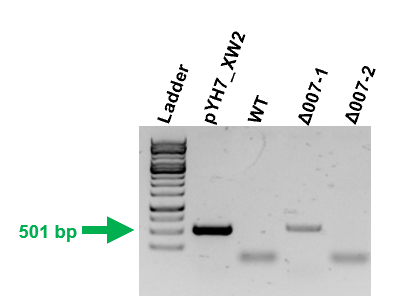 | Knock out of XW1 strain BGC007 using pYH7_XW2 plasmid generating XW2 strain (Δ006Δ007). Primers used for homology arms: pYH7_XW2_L_F/pYH7_XW2_L_R and pYH7_XW2_R_F/pYH7_XW2_R_F. Validated using primers: XW2_CK_F/XW2_CK_R |
| 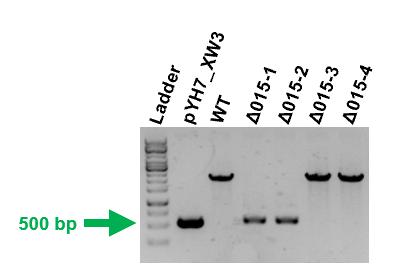 | Knock out of XW2 strain BGC015 using pYH7_XW3 plasmid generating XW3 strain (Δ006Δ007Δ015). Primers used for homology arms: pYH7_XW3_L_F/pYH7_XW3_L_R and pYH7_XW3_R_F/pYH7_XW3_R_F. Validated using primers: XW3_CK_F/XW3_CK_R |
| 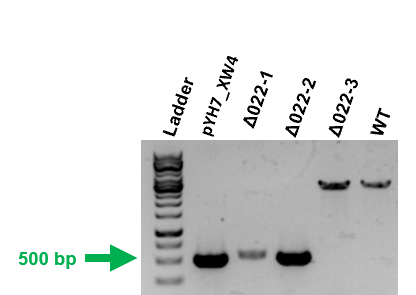 | Knock out of XW3 strain BGC022 using pYH7_XW4 plasmid generating XW4 strain (Δ006Δ007Δ015Δ022). Primers used for homology arms: pYH7_XW4_L_F/pYH7_XW4_L_R and pYH7_XW4_R_F/pYH7_XW4_R_F. Validated using primers: XW4_CK_F/XW4_CK_R |
| 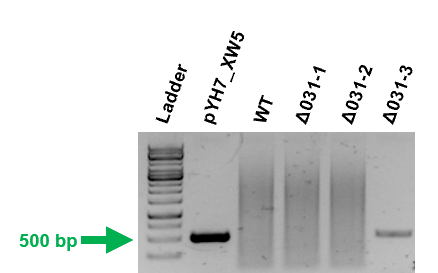 | Knock out of XW4 strain BGC031 using pYH7_XW5 plasmid generating XW5 strain (Δ006Δ007Δ015Δ022Δ031). Primers used for homology arms: pYH7_XW5_L_F/pYH7_XW5_L_R and pYH7_XW5_R_F/pYH7_XW5_R_F. Validated using primers: XW5_CK_F/XW5_CK_R |
| 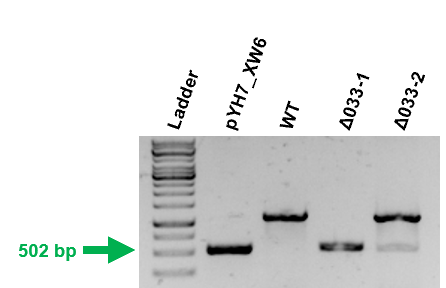 | Knock out of XW5 strain BGC033 using pYH7_XW6 plasmid generating XW6 strain (Δ006Δ007Δ015Δ022Δ031Δ033). Primers used for homology arms: pYH7_XW6_L_F/pYH7_XW6_L_R and pYH7_XW6_R_F/pYH7_XW6_R_F. Validated using primers: XW6_CK_F/XW6_CK_R |
| 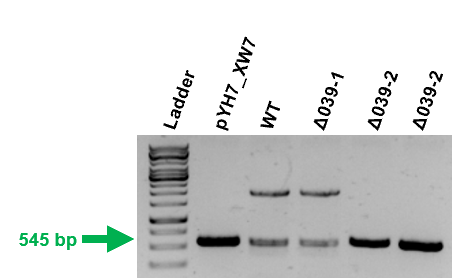 | Knock out of XW6 strain BGC039 using pYH7_XW7 plasmid generating XW7 strain (Δ006Δ007Δ015Δ022Δ031Δ033Δ039). Primers used for homology arms: pYH7_XW7_L_F/pYH7_XW7_L_R and pYH7_XW7_R_F/pYH7_XW7_R_F. Validated using primers: XW7_CK_F/XW7_CK_R |
| 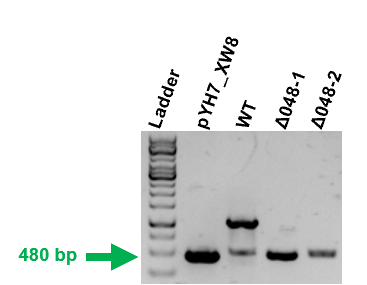 | Knock out of XW7 strain BGC039 using pYH7_XW8 plasmid generating XW8 strain (Δ006Δ007Δ015Δ022Δ031Δ033Δ039Δ048). Primers used for homology arms: pYH7_XW8_L_F/pYH7_XW8_L_R and pYH7_XW8_R_F/pYH7_XW8_R_F. Validated using primers: XW8_CK_F/XW8_CK_R |
| 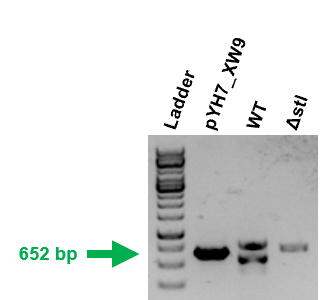 | Knock out of XW8 strain BGC039 using pYH7_XW9 plasmid generating XW9 – CH strain (Δ006Δ007Δ015Δ022Δ031Δ033Δ039Δ048Δstl). Primers used for homology arms: pYH7_XW9_L_F/pYH7_XW9_L_R and pYH7_XW9_R_F/pYH7_XW9_R_F. Validated using primers: XW9_CK_F/XW9_CK_R |

**Table S3**. Oligonucleotide primers used in this study.

| **Primer** | **Sequence (5’ to 3’)** |
| --- | --- |
| **Knock out** | |
| pYH7_XW1_L_F | TGATCAAGGCGAATACTTCAGTCGTCGTGTTCGTGCAGTGGGCACG |
| pYH7_XW1_L_R | AGATCATGGCGCACTCAGACCCGGA |
| pYH7_XW1_R_F | GTCTGAGTGCGCCATGATCTGGGGCA |
| pYH7_XW1_R_R | CGTCGACCTGCAGGCATGCAGGTCACCCTCGTCTTCCTTCCGGCCAAGGACA |
| PYH7_XW2_L_F | TGATCAAGGCGAATACTTCACATCCCGACCTCGAGCCCC |
| PYH7_XW2_L_R | AGCTGGCGCGGCGCCGGCGAGCT |
| PYH7_XW2_R_F | TCGCCGGCGCCGCGCCAGCTCG |
| PYH7_XW2_R_R | CGTCGACCTGCAGGCATGCAGCAGCCACAGCTGGGC |
| PYH7_XW3_L_F | TGATCAAGGCGAATACTTCACCGCCGTGCCGTCCG |
| PYH7_XW3_L_R | CCAGCGGCGGCGCCCTCTCCCAGCACCG |
| PYH7_XW3_R_F | GGAGAGGGCGCCGCCGCTGGGATGAAG |
| PYH7_XW3_R_R | CGTCGACCTGCAGGCATGCATCGCTGAGCACGGTCTTG |
| PYH7_XW4_L_F | TGATCAAGGCGAATACTTCAGAGCATGAGCGTGGGGAC |
| PYH7_XW4_L_R | GTCGGGCAGGCACCTCCATGCCCGGAC |
| PYH7_XW4_R_F | CATGGAGGTGCCTGCCCGACGGCTACGA |
| PYH7_XW4_R_R | CGTCGACCTGCAGGCATGCATCCTTACGGAACGTGCCCA |
| PYH7_XW5_L_F | TGATCAAGGCGAATACTTCAGCCGCCGGTCCTCGGGCG |
| PYH7_XW5_L_R | GCCGCAAGAACCGCGTCCCGGTGC |
| PYH7_XW5_R_F | CGGGACGCGGTTCTTGCGGCCGGTGATGGTGA |
| PYH7_XW5_R_R | CGTCGACCTGCAGGCATGCAACGCCGTCCGCACCAA |
| PYH7_XW6_L_F | TGATCAAGGCGAATACTTCACTCGTGGGCTTCGTCTTCCT |
| PYH7_XW6_L_R | TTTCCCAGCGCGTGGTTGTCCTGGTGATCGCCTA |
| PYH7_XW6_R_F | GACAACCACGCGCTGGGAAACGGTGGATAT |
| PYH7_XW6_R_R | CGTCGACCTGCAGGCATGCATCGTGTCCTTGCCGTCGT |
| PYH7_XW7_L_F | TGATCAAGGCGAATACTTCACCCGCAGCACGCCC |
| PYH7_XW7_L_R | CCGACCGGGTGCCGCCGCCGACTTC |
| PYH7_XW7_R_F | CGGCGGCGGCACCCGGTCGGAGTT |
| PYH7_XW7_R_R | CGTCGACCTGCAGGCATGCACGTACTGGTCGATCTCGCCCT |
| PYH7_XW8_L_F | TGATCAAGGCGAATACTTCAGATCGATGTGCACGGTGCGC |
| PYH7_XW8_L_R | GACCAGATGAACCTCCAACAGGATGAGTCCGG |
| PYH7_XW8_R_F | TGTTGGAGGTTCATCTGGTCGAAGCCGC |
| PYH7_XW8_R_R | CGTCGACCTGCAGGCATGCAACTCGTTGCAGGAGCAATCAC |
| PYH7_XW9_L_F | GATCAAGGCGAATACTTCATGGAGCGTCCGGTGCC |
| PYH7_XW9_L_R | CCTTGAGTTCAGAAATCCGACGGGCTTGGCG |
| PYH7_XW9_R_F | CGGATTTCTGAACTCAAGGCCAACGGGCT |
| PYH7_XW9_R_R | ACCTGCAGGCATGCATGGTCTCCGGACGATTCGACGAG |
| **Knock out screening** | |
| XW1_CK_F | TACCTGGAACGAGTCGCCC |
| XW1_CK_R | TTCACCCGCCAGCGGTGCG |
| XW2_CK_F | AACACCGCGTGCGGGCCCTC |
| XW2_CK_R | CGTGCCGGTCACCAGGACG |
| XW3_CK_F | AGGACCCGGCATCGGCCTGG |
| XW3_CK_R | GCGGCGCCACGGACCTGCC |
| XW4_CK_F | TTCAGGACAGGCCCAGTTCG |
| XW4_CK_R | GCGATGACGAAGCTGGTGCG |
| XW5_CK_F | CATGGTGGGCACCGCGGGTC |
| XW5_CK_R | CCGCCGCCGCCACCATCGTC |
| XW6_CK_F | ACGAGCGAGGCAAGGAAGAG |
| XW6_CK_R | CCGCGGATCCATGGCGCGTG |
| XW7_CK_F | ACCTGGTCGTCGGCTCGCTG |
| XW7_CK_R | TCCGGTCGGCCTTGGCGTTG |
| XW8_CK_F | TAGTCGATCTGGTCGTAGCCGTC |
| XW8_CK_R | CACCACGCCTCCCGGTCGG |
| XW9_CK_F | GATCATCATCGGCAGCATC |
| XW9_CK_R | CTGGAGCACCAGATCATCC |
| **Screening for conjugated BGCs** | |
| Ery_F | CGAGATGATTCCGTTGGTG |
| Ery_R | CCATCGACACATTGATGACC |
| Act_F | AAGGAGCTGTTCGGATTGAA |
| Acr_R | GAGGTGAGCAGTTCCCAGAA |
| Bipent_F1 | ACCATGCCGATCAGGTAGAC |
| Bipent_R1 | GTGCAGAACTCCGTCGAGAT |
| Aur1_F | CGGAATGGGACGTGAACTGT |
| Aur1_R | GGGGCTCAGCTTCCAGAATT |
| EryAIII_F | TTTTCGTCTTCCCAGGTCAGG |
| EryAIII_R | GACACCATCACGGAGAACAACA |
| EryAII_F | GATCTGCTGGAATCCTCCGAG |
| EryAII_R | CGAGACCATGATCGAGAAGAGC |
| EryF_F | GATCTCGAAAGCGACTCCTTCC |
| EryF_R | CATGTTGGTGGCGAAGTAGTTC |
| EryCIV_F | GTCATGCCTTCGATGACGTTC |
| EryCIV_R | AAGAAGAGTTTGACCTGGTGCT |
| **Auroramycin BGC cloning** | |
| pBE45-R | ATCTTTATAGTCCTGTCGGGTTTCG |
| pBE48-F | TTACCAATGCTTAATCAGTGAGGCACC |
| pBE48-auro-large-R | CACTTGCTGTGTTTACGCCGTGTTGCGAACTTCTCACCGACAGCGACACACTTGCATCG |
| pBE45-auro-large-F | TGCGTGTTGTGCTTCACACAATCACCAGAATCGGAACTAGACGCTCAGTGGAACGAAAAC |
| auro-large-gRNA1-F | AATTAATACGACTCACTATAGGGAATTTCTACTGTTGTAGATCGCCGTGTTGCGAACTTC |
| auro-large-gRNA1-R | GAAGTTCGCAACACGGCGATCTACAACAGTAGAAATTCCCTATAGTGAGTCGTATTAATT |
| auro-large-gRNA2-F | AATTAATACGACTCACTATAGGGAATTTCTACTGTTGTAGATACACAATCACCAGAATCG |
| auro-large-gRNA2-R | CGATTCTGGTGATTGTGTATCTACAACAGTAGAAATTCCCTATAGTGAGTCGTATTAATT |

**Table S4**. Parameters for evaluation of strain performance in this study. Scores were assigned based on the ability of strain to meet the criteria efficiently and based on the data from **Fig. 1**, **Fig. 2**, **Fig. 4** as well as previously published data. Highest score (5) was given to the strain that is either best performer or meets all necessary requirements. The lowest score (0) was given to strains that do not meet any of the requirements. The production was divided into 5 bands and depending on the production level score was assigned accordingly (i.e., top band – 5, followed by 4, 3, 2, 1 and 0 if no production observed) based on **Fig. S18**. Currently published genetic tools were evaluated according to criteria such as: attB sites, CRISPR editing tools, other genome editing strategies, established promoter and regulatory toolbox. Growth rate and biomass accumulation were evaluated and compared against the most productive strain. Note that *S. venezuelae* NRRL B-65442 strain was not compared due to issues with collecting enough spores for experiment. Phylogenetics was evaluated by how distinctive the strains are from each other based on maximum percent identity (MPI). Full description and scoring values are included in Table S6. Highly related strains were scored as 1 and most phylogenetically unrelated strains will be scored as 5. For WT and CH strains the diversity rate was not impacted by their identical proximity. Sporulation was evaluated based on the CFU mL^-1^ values from a single plate (further explained and described in Fig S19).

|  | **WT** | **CH** | **TK24** | **M1152** | **J1074** | **B-65442** |
| --- | --- | --- | --- | --- | --- | --- |
| Phylogenetics | 2 | 2 | 1 | 1 | 4 | 3 |
|  |  |  |  |  |  |  |
| Growth | 4 | 5 | 4 | 3 | 3 | 0 |
| Biomass | 5 | 5 | 3 | 3 | 4 | 0 |
| Sporulation | 5 | 5 | 5 | 5 | 5 | 4 |
| Genetic tools | 2 | 2 | 5 | 5 | 5 | 4 |
|  |  |  |  |  |  |  |
| Diversity (R5) Bipentaromycin | 1 | 5 | 2 | 3 | 4 | 0 |
| Diversity (SFM) Bipentaromycin | 5 | 5 | 1 | 2 | 2 | 0 |
|  |  |  |  |  |  |  |
| Bipentaromycin (R5) | 2 | 3 | 1 | 1 | 5 | 0 |
| Bipentaromycin (SFM) | 4 | 5 | 1 | 1 | 1 | 0 |
|  |  |  |  |  |  |  |
| Actinorhodin (R5) | 2 | 5 | 2 | 5 | 3 | 0 |
| Actinorhodin (SFM) | 1 | 2 | 1 | 5 | 2 | 1 |
|  |  |  |  |  |  |  |
| Erythromycin (R5) | 0 | 5 | 0 | 0 | 0 | 1 |
| Erythromycin (SFM) | 5 | 5 | 0 | 0 | 0 | 1 |
|  |  |  |  |  |  |  |
| Auroramycin (R5) | 0 | 1 | 0 | 5 | 2 | 0 |
| Auroramycin (SFM) | 0 | 5 | 0 | 1 | 3 | 0 |

**Table S5**. All identified bipentaromycin derivatives with their respective mass, positive and negative m/z. Number in brackets indicates peaks based on **Fig. S7** chromatogram b.

|  | **mass** | **Negative ion** | **Positive ion** |
| --- | --- | --- | --- |
| **Bipentaromycin A (1)** | 708 | 707 | 709 |
| **Bipentaromycin B (2)** | 706 | 705 | 707 |
| **Bipentaromycin C (3)** | 750 | 749 | 751 |
| **Bipentaromycin D (4)** | 748 | 747 | 749 |
| **Bipentaromycin E (5)** | 778 | 777 | 779 |
| **Bipentaromycin F (6)** | 776 | 775 | 777 |
| **Bipentaromycin G (7)** | 792 | 791 | 793 |
| **Bipentaromycin H (8)** | 790 | 789 | 791 |

**Table S6**. Percent identity matrix for 16S rRNA gene sequences of model strains. To calculate the phylogenetic score of the model strains investigated, a multiple sequence alignment of their 16S rRNA gene sequences was constructed, from which a percent identity matrix was obtained. The non-self maximum percent identity (MPI) values, which reflects the maximum relatedness to other strains in the group, are highlighted in each row. Strains with a higher MPI receive a low score, while rows with a lower MPI receive a high score.

| Strain | Percent identity | | | | | | Score |
| --- | --- | --- | --- | --- | --- | --- | --- |
|  | J1074 | TK24 | A3(2) | B-65442 | A4420 | MA-4680 |  |
| *S. albus* J1074 | 100 | 96.22 | 96.29 | 95.88 | 96.12 | 95.21 | 4 |
| *S. lividans* TK24 | 96.22 | 100 | 99.8 | 96.35 | 96.98 | 97.14 | 1 |
| *S. coelicolor* A3(2) | 96.29 | 99.8 | 100 | 96.55 | 96.9 | 97.35 | 1 |
| *S. venezuelae* NRRL B-65442 | 95.88 | 96.35 | 96.55 | 100 | 98.19 | 97.68 | 3 |
| *Streptomyces* sp. A4420 | 96.12 | 96.98 | 96.9 | 98.19 | 100 | 98.62 | 2 |
| *S. avermitilis* MA-4680 | 95.21 | 97.14 | 97.35 | 97.68 | 98.62 | 100 | 2 |


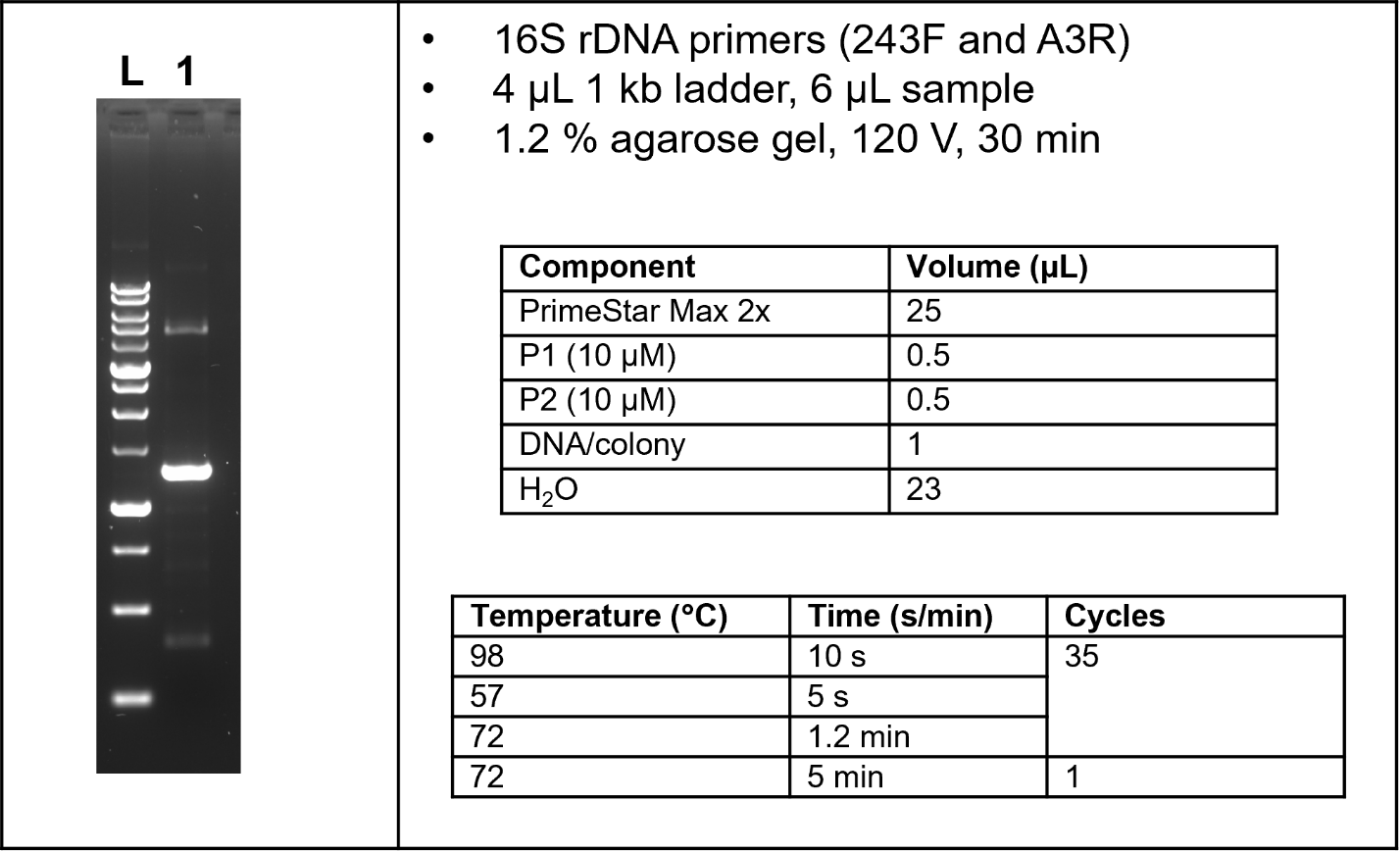


**Fig. S1**. Amplification of 16S rDNA from *Streptomyces* sp. A4420. Purified gDNA was used to amplify and sequence 16S rDNA. The composition of PCR mixture and protocol are stated in the tables within the figure. PrimeSTAR Max (Takara Bio, Japan) polymerase and 243F/A3R primer pair was used and expected size band obtained for sequencing.


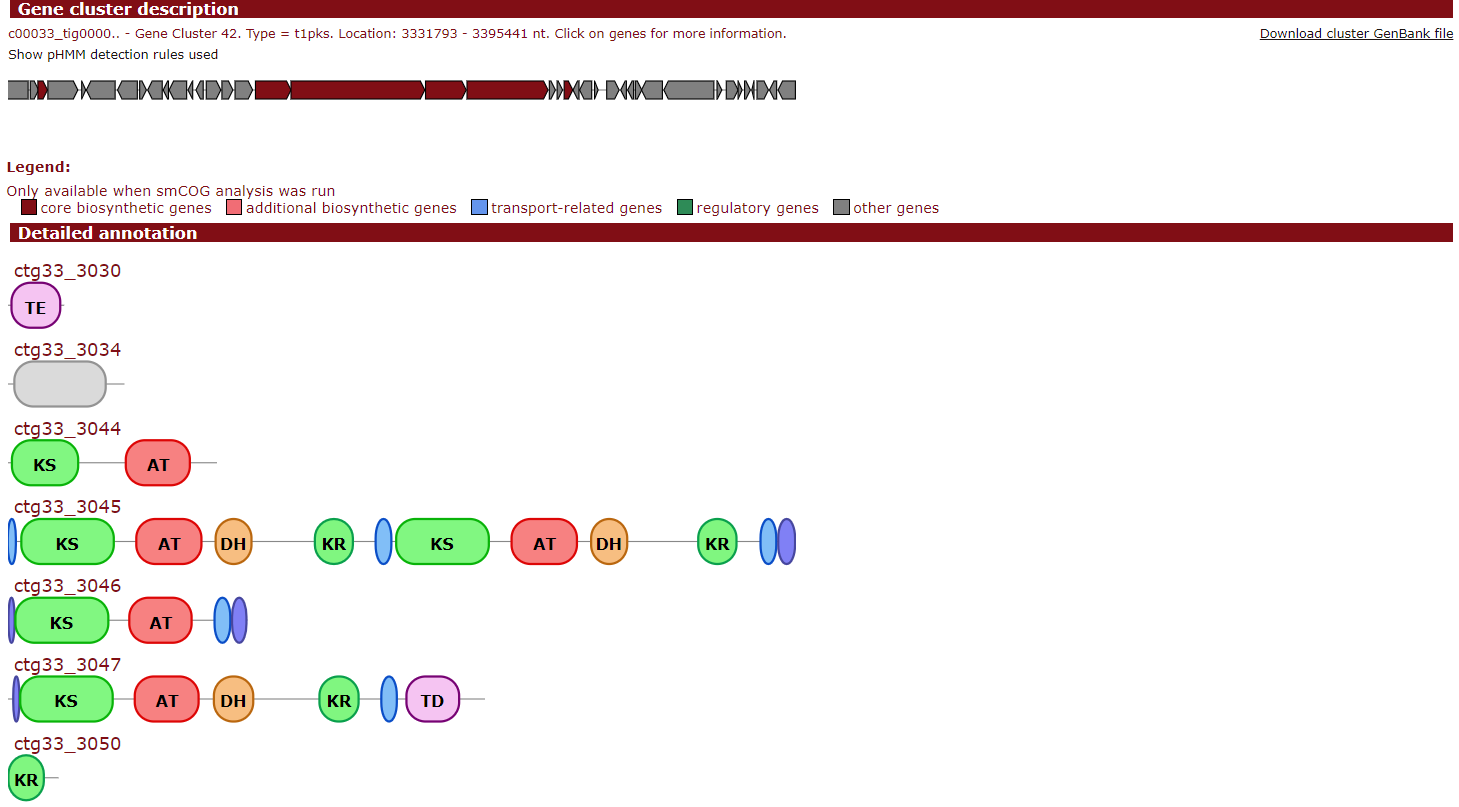


**Fig. S2**. AntiSMASH identification and annotation of streptazolin BGC from *Streptomyces* sp. A4420 genomic data (indicated as streptazone E cluster with 83 % similarity score). Compared to streptazone E cluster [9], highlighted BGC lacks single module, matching the expected elongation of streptazolin. Additional signature genetic elements for piperidine bacterial alkaloids including trans-aminase and TR domain (instead of typical TE domain) are present in this BGC.

**Fig. S3**. BPC positive mode (a), extracted ion chromatograms (EIC) at m/z 734 (b) and m/z analysis (c) for erythromycin standard in MeOH. Samples analyzed using 30 min gradient method with Bruker amaZon SL system. The peak for erythromycin elutes at 12.3 min (labeled with asterisk). Mass spectra (**c**) is shown for erythromycin peak at 12.3 min: *m/z* 734.47 [*M*+H]^+^ (calculated 734.9 [*M*+H]^+^). The expected value based on literature matches with erythromycin standard sample tested.

**Fig. S4**. BPC positive mode (a), extracted ion chromatograms (EIC) at m/z 734 (b) and m/z analysis (c) for heterologous erythromycin production in CH strain. Samples analyzed using 30 min gradient method with Bruker amaZon SL system. The peak for erythromycin elutes at 12.3 min (labeled with asterisk) matching with **Fig. S3** erythromycin standard. Mass spectra (**c**) is shown for erythromycin peak at 12.3 min: *m/z* 734.38 [*M*+H]^+^ (calculated 734.9 [*M*+H]^+^). The expected erythromycin m/z value based on literature and previously tested standard **Fig. S3a** matches with heterologously expressed BGC.

**Fig. S5**. BPC positive mode (a), extracted ion chromatograms (EIC) at m/z 397 (b) and m/z analysis (c) for auroramycin fermentation sample from *S. roseosporus* NRRL 15 998 Rose22 strain used as a standard. Samples analyzed using 30 min gradient method with Bruker amaZon SL system. The peak for auroramycin elutes at 13.9 min (labeled with asterisk). Based on the literature two main ionized species are present for auroramycin: *m*/*z* 794.5319 [*M*+H]^+^ and 397.7696 [*M*+2 H]^2+^. The latter shows higher levels of ionization and was selected for EIC. Mass spectra (**c**) is shown for auroramycin peak at 13.9 min: *m/z* 794.46 [*M*+H]^+^ and 397.70 [*M*+2 H]^2+^ (HRMS determined and published *m*/*z* 794.5319 [*M*+H]^+^ and 397.7696 [*M*+2 H]^2+^). The expected value based on literature matches with auroramycin producing *S. roseosporus* NRRL 15 998 Rose22 strain.

**Fig. S6**. BPC positive mode (a), extracted ion chromatograms (EIC) at m/z 397 (b) and m/z analysis (c) for heterologous auroramycin production in CH strain. Samples analyzed using 30 min gradient method with Bruker amaZon SL system. The peak for auroramycin elutes at 13.9 min (labeled with asterisk). Based on the literature two main ionized species are present for auroramycin: *m*/*z* 794.5319 [*M*+H]^+^ and 397.7696 [*M*+2 H]^2+^. The latter shows higher levels of ionization and was selected for EIC. The peak for auroramycin elutes at 13.9 min matching with **Fig. S5** auroramycin producing standard. Mass spectra (**c**) is shown for auroramycin peak at 13.9 min: *m/z* 794.49 [*M*+H]^+^ and 397.74 [*M*+2 H]^2+^ (HRMS determined and published *m*/*z* 794.5319 [*M*+H]^+^ and 397.7696 [*M*+2 H]^2+^). The expected auroramycin m/z value based on literature and previously tested standard **Fig. S5** matches with heterologously expressed BGC.

**Fig. S7**. LC-MS analysis of heterologous bipentaromycin BGC expression in CH strain. A280 absorbance was measured for empty CH strain (a) and heterologously expressed bipentaromycin BGC (b) fermentation samples using 30 min gradient method with Bruker amaZon SL system. Peaks 1 – 8 were identified to be bipentaromycin derivatives and their molecular mass properties stated in **Table S5**.

**Fig. S8**. Mass spectra for bipentaromycin A (peak 1) at 18.4 min based on **Fig. S7b**: *m/z* 709.04 [*M*+H]^+^ (**a**) and 707.11 [*M*-H]^-^ (**b**). Expected ionization was validated using previously published data [1].

**Fig. S9**. Mass spectra for bipentaromycin B (peak 2) at 18.8 min based on **Fig. S7b**: *m/z* 707.02 [*M*+H]^+^ (**a**) and 705.10 [*M*-H]^-^ (**b**). Expected ionization was validated using previously published data [1].

**Fig. S10**. Mass spectra for bipentaromycin C (peak 3) at 19.9 min based on **Fig. S7b**: *m/z* 751.07 [*M*+H]^+^ (**a**) and 749.14 [*M*-H]^-^ (**b**). Expected ionization was validated using previously published data [1].

**Fig. S11**. Mass spectra for bipentaromycin D (peak 4) at 20.2 min based on **Fig. S7b**: *m/z* 749.09 [*M*+H]^+^ (**a**) and 747.10 [*M*-H]^-^ (**b**). Expected ionization was validated using previously published data [1].

**Fig. S12**. Mass spectra for bipentaromycin E (peak 5) at 21.5 min based on **Fig. S7b**: *m/z* 779.18 [*M*+H]^+^ (**a**) and 777.18 [*M*-H]^-^ (**b**). Expected ionization was validated using previously published data [1].

**Fig. S13**. Mass spectra for bipentaromycin F (peak 6) at 21.8 min based on **Fig. S7b**: *m/z* 777.13 [*M*+H]^+^ (**a**) and 775.15 [*M*-H]^-^ (**b**). Expected ionization was validated using previously published data [1].

**Fig. S14**. Mass spectra for bipentaromycin G (peak 7) at 22.0 min based on **Fig. S7b**: *m/z* 793.10 [*M*+H]^+^ (**a**) and 791.21 [*M*-H]^-^ (**b**). Expected ionization was validated using previously published data [1].

**Fig. S15**. Mass spectra for bipentaromycin H (peak 8) at 22.2 min based on **Fig. S7b**: *m/z* 791.11 [*M*+H]^+^ (**a**) and 789.16 [*M*-H]^-^ (**b**). Expected ionization was validated using previously published data [1].


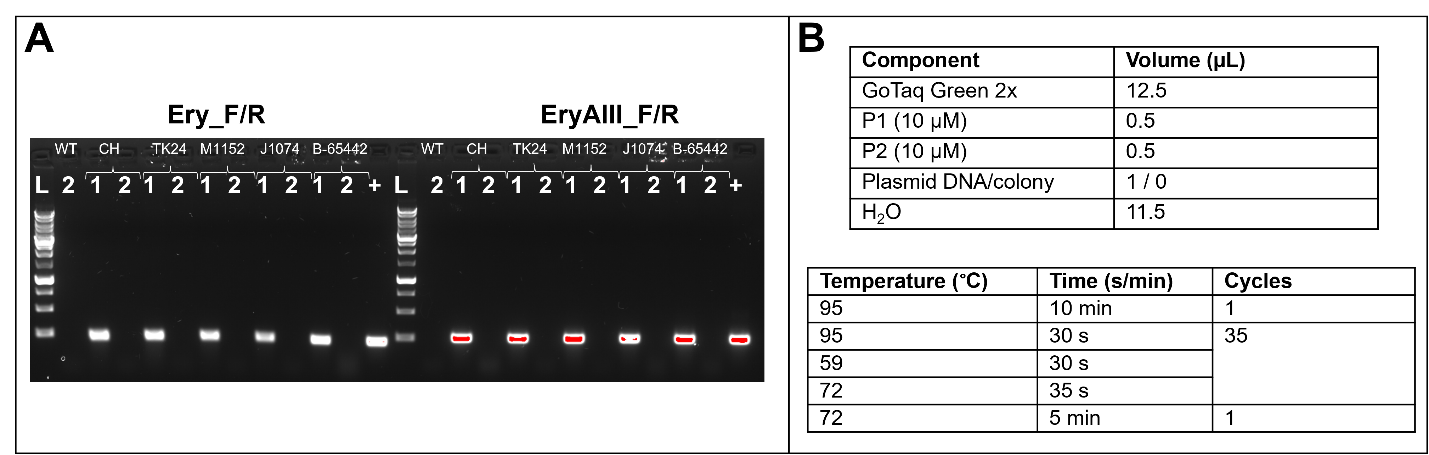


**Fig. S16**. Agarose gel for validating presence of intact erythromycin BGC using primer pairs Ery_F/R and EryAIII_F/R. Gel A shows PCR analysis of amplified fragments using setting in figure B. As a control WT empty strain, CH empty and containing erythromycin BGC and pBE plasmid containing erythromycin cluster (+) were used. Primer pairs are stated above each set of samples with L (ladder – 1 kb), 1 (exconjugant strain containing BGC) and 2 (empty strain).


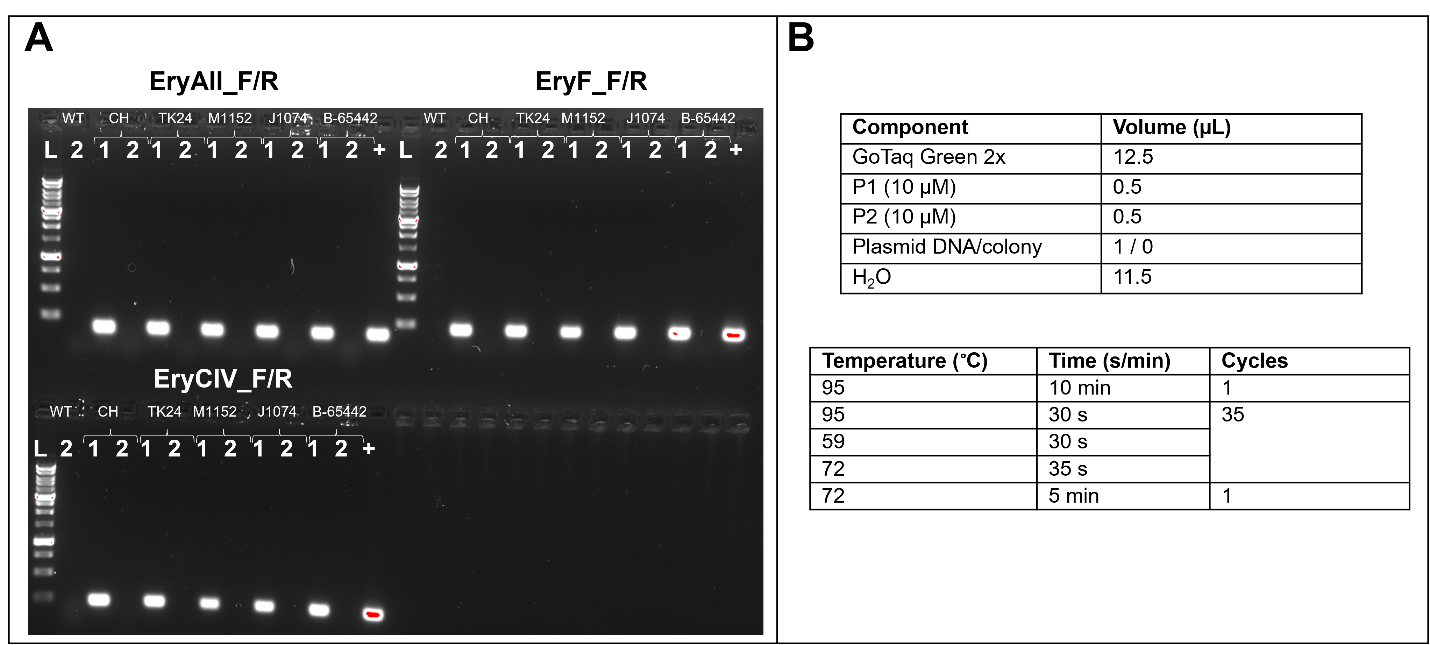


**Fig. S17**. Agarose gel for validating presence of intact erythromycin BGC using primer pairs EryAII_F/R, EryF_F/R and EryCIV_F/R. Gel A shows PCR analysis of amplified fragments using setting in figure B. As a control WT empty strain, CH empty and containing erythromycin BGC and pBE plasmid containing erythromycin cluster (+) were used. Primer pairs are stated above each set of samples with L (ladder – 1 kb), 1 (exconjugant strain containing BGC) and 2 (empty strain).


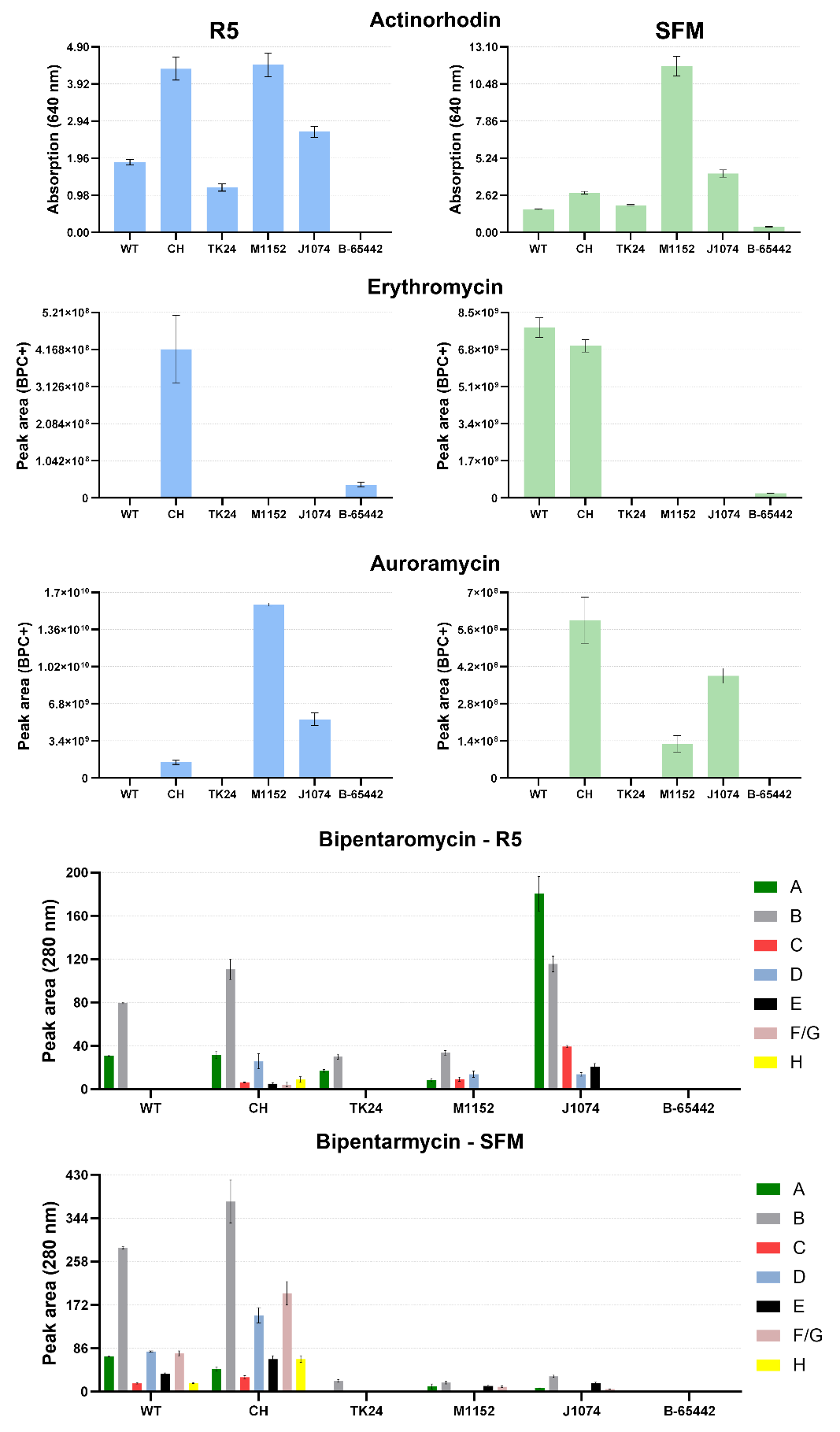


**Fig. S18**. Heterologous production level of actinorhodin, erythromycin, auroramycin and bipentaromycin in *Streptomyces* sp. A4420 (WT), *Streptomyces* sp. A4420 CH, *S. lividans* TK24, *S. coelicolor* M1152, *S. albus* J1074, *S. venezuelae* NRRL B-65442 in R5 (blue) and SFM (green) media. Bipentaromycin congeners A – H are highlighted in different colors. Fermentation media and investigated metabolites are indicated above. To facilitate comparison, the production scale is normalized to the highest producer for each condition and the production figures are split into 5 equal bands (dashed horizontal grey lines). All production levels are represented with a linear scale, using A640 absorption (actinorhodin), peak area in BPC+ mode (erythromycin and auroramycin) and peak area using A280 absorption (bipentaromycin). This figure acts as extension for **Fig. 4**, including presence of bands for production evaluation, split evaluation of actinorhodin, erythromycin and auroramycin, and absolute units incorporated for y axis.


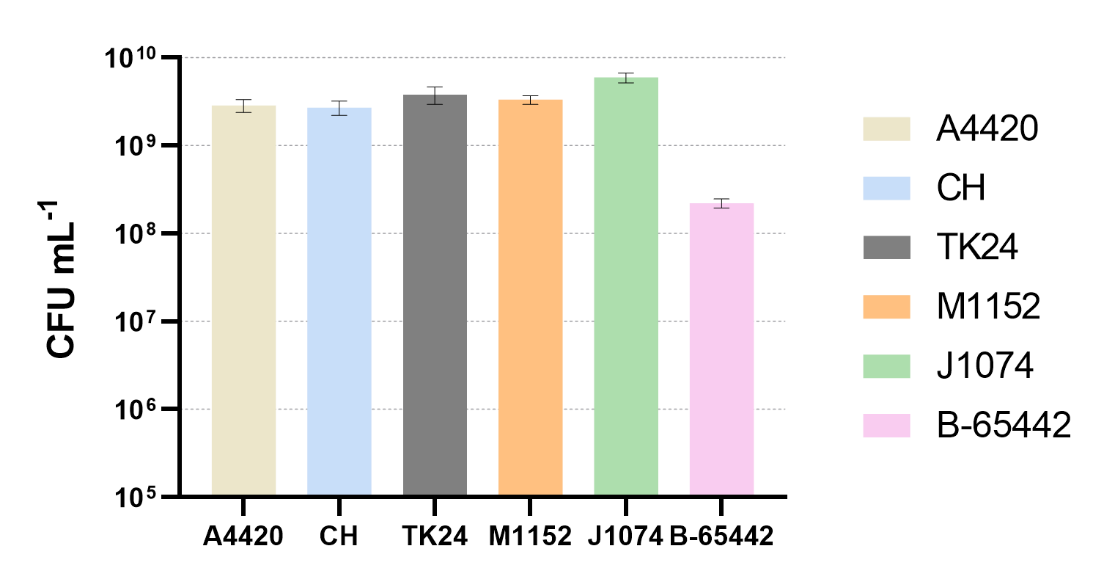


**Fig. S19**. Sporulation score evaluated based on colony forming unit (CFU) measurement. The score was assigned based on the band of the CFU mL^-1^ with cut off being 10^5^, where above 10^9^ CFU mL^-1^ was scored 5, between 10^8^ and 10^9^ CFU mL^-1^ was scored 4 and so on. A single plate containing SFM agar was inoculated with spore stocks of each individual strains separately. Spores were evenly distributed to cover the whole plate and incubated at 30 °C for 7 days. Each spore preparation was performed in triplicates. After incubation period, spores we collected by flooding the plate with 2 mL 50 mM Tris-HCl pH 7.4, 0.001 % triton X-100 and gently scraping the surface with a glass slide followed by additional 2 mL wash. Mycelia was filtered out using cotton balls and spores centrifuged. Each spore stock collected from an individual plate was resuspended in 1 mL 20% glycerol and used for serial dilution to calculate CFU mL^-1^ on SFM plates at 30 °C after 3 days of growth. Only countable plates (30 – 300 colonies per plate) were used for final CFU mL^-1^ evaluation.

**References**

1. Enghiad B, Huang C, Guo F, Jiang G, Wang B, Tabatabaei SK, Martin TA, Zhao H: Cas12a-assisted precise targeted cloning using in vivo Cre-lox recombination. Nat Commun. 2021;12(1):1171.

2. Mazodier P PR, Thompson C: Intergeneric conjugation between Escherichia coli and Streptomyces species. J Bacteriol. 1989;171(6):3583-5.

3. Ruckert C, Albersmeier A, Busche T, Jaenicke S, Winkler A, Friethjonsson OH, Hreggviethsson GO, Lambert C, Badcock D, Bernaerts K, et al: Complete genome sequence of Streptomyces lividans TK24. J Biotechnol. 2015;199:21-2.

4. Gomez-Escribano JP, Bibb MJ: Engineering Streptomyces coelicolor for heterologous expression of secondary metabolite gene clusters. Microb Biotechnol. 2011;4(2):207-15.

5. Som NF, Heine D, Holmes NA, Munnoch JT, Chandra G, Seipke RF, Hoskisson PA, Wilkinson B, Hutchings MI: The Conserved Actinobacterial Two-Component System MtrAB Coordinates Chloramphenicol Production with Sporulation in Streptomyces venezuelae NRRL B-65442. Front Microbiol. 2017;8:1145.

6. Zaburannyi N RM, Ostash B, Fedorenko V, Luzhetskyy A: Insights into naturally minimised Streptomyces albus J1074 genome. BMC Genomics. 2014;15:97.

7. Lim YH, Wong FT, Yeo WL, Ching KC, Lim YW, Heng E, Chen S, Tsai DJ, Lauderdale TL, Shia KS, et al: Auroramycin: A Potent Antibiotic from Streptomyces roseosporus by CRISPR-Cas9 Activation. Chembiochem. 2018.

8. He Q, Li L, Yang T, Li R, Li A: Functional Characterization of a Ketoreductase-Encoding Gene med-ORF12 Involved in the Formation of a Stereospecific Pyran Ring during the Biosynthesis of an Antitumor Antibiotic Medermycin. PLoS One. 2015;10(7):e0132431.

9. Ohno S, Katsuyama Y, Tajima Y, Izumikawa M, Takagi M, Fujie M, Satoh N, Shin-Ya K, Ohnishi Y: Identification and Characterization of the Streptazone E Biosynthetic Gene Cluster in Streptomyces sp. MSC090213JE08. Chembiochem. 2015;16(16):2385-91.
